# Ubiquitin and ubiquitin‐like modifications in the endoplasmic reticulum stress response

**DOI:** 10.1111/febs.70622

**Published:** 2026-06-16

**Authors:** Tony Avril, Matthieu Le Gallo, Elodie Lafont

**Affiliations:** ^1^ INSERM U1242 University of Rennes Rennes France; ^2^ Centre de lutte contre le cancer Eugène Marquis Rennes France

**Keywords:** endoplasmic reticulum, ER stress, ERAD, ER‐ribosomal quality control, integrated stress response, ubiquitin, ubiquitin‐like, unfolded protein response

## Abstract

The endoplasmic reticulum (ER) is a cellular organelle frequently subjected to stress under both physiological and pathological circumstances, associated with the accumulation of mis/unfolded proteins in its lumen. To cope with this stress, cells have evolved an adaptive program called the unfolded protein response (UPR), whose primary function is to restore ER proteostasis. When the stress is prolonged, the UPR can also trigger cell death. The UPR controls multiple machineries involved in pre‐emptive quality control (QC) of proteins prior to ER entry, ribosome‐associated QC, protein folding within the ER, protein degradation through various processes, and export from the ER for secretion. Because the UPR and the machineries it controls play fundamental roles in determining cell fate, they are finely regulated, including through post‐translational modifications (PTMs). In this review, we focus on the role of the ubiquitin and ubiquitin‐like PTMs in the regulation and mediation of ER proteostasis. We specifically focus on three core processes: the UPR, ER‐associated ribosome QC and ER‐associated degradation. Lastly, we briefly discuss how Ub and Ubl also control the integrated stress response and the formation of inter‐organelle membrane contact sites and thus act as general regulators of responses to cellular stresses beyond ER proteotoxicity.

## Graphical abstract

Endoplasmic reticulum (ER) stress activates various proteostasis control processes, including the unfolded protein response, ribosome‐associated quality control, and ER‐associated degradation. Ubiquitin and ubiquitin‐like modifications dynamically regulate these processes to determine cell fate, promoting adaptation or inducing cell death.

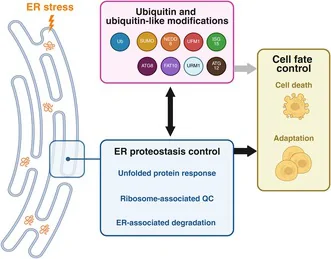

**Abbreviations**
40S/60S/80S
: Ribosomal subunit
ANKZF1
: Ankyrin repeat and zinc finger peptidyl transfer RNA hydrolase 1
ASCC
: Activating signal cointegrator 1 complex
ASK1
: Apoptosis signal‐regulating kinase 1
ATF4/6
: Activating transcription factor 4/6
ATG16L1
: ATG16 like 1
ATG3/5/7/8/10/12
: Autophagy‐related 3/5/7/8/10/12
AUP1
: Ancient ubiquitous protein 1
BAG6
: BAG co‐chaperone 6
BAK
: BCL2‐agonist/killer
BARD1
: BRCA1‐associated RING domain 1
BAX
: BCL2‐associated X, apoptosis regulator
BCL2
: B‐cell cll/lymphoma 2
BIM
: BCL2‐interacting mediator of cell death
BiP
: Binding‐immunoglobulin protein
BRCA1
: Breast cancer DNA repair associated 1
bTrCP
: Beta‐transducin repeat containing E3 ubiquitin protein ligase
CAT
: C‐terminal alanine‐threonine
CDK5RAP3
: Cyclin‐dependent kinase 5 regulatory subunit‐associated protein 3
CFTR
: Cystic fibrosis transmembrane conductance regulator
CGRRF1
: Cell growth regulator with ring finger domain 1
CHIP
: Carboxy terminus of HSP70‐interacting protein
ChIP
: Chromatin immunoprecipitation
CHOP
: CCAAT/enhancer‐binding protein homologous protein
cIAP1/2
: Cellular inhibitor of apoptosis protein 1/2
CREP
: Constitutive repressor of eIF2α phosphorylation
CTSB
: Cathepsin B
CYB5R3
: Cytochrome B5 reductase 3
DDRGK1
: DDRGK domain‐containing 1
DERLIN1/2
: Der1‐like domain family, member 1/2
DRP1
: Dynamin‐related protein 1
EDEM1/2/3
: ER degradation‐enhancing alpha‐mannosidase‐like protein 1/2/3
EIF2AK3
: eIF2α kinase 3
eIF2α
: Eukaryotic translation initiation factor 2 alpha
ER
: Endoplasmic reticulum
ERAD
: ER‐associated degradation
ERDJ5
: Endoplasmic reticulum DNA J domain‐containing protein 5
eRF1
: Eukaryotic peptide chain release factor 1
ERGIC
: ER‐Golgi intermediate compartment
ERLAD
: ER‐lysosomal degradation
ERLIN1/2
: ER lipid raft associated 1/2
ERO1α
: Endoplasmic reticulum oxidoreductin 1α
ERP18/46
: ER protein 18/46
ER‐RQC
: ER‐ribosomal‐associated quality control
eS10
: Eukaryotic small ribosomal subunit
FAM134B
: Family with sequence similarity 134 member B
FAT10
: HLA‐F adjacent transcript 10
FBW7
: F‐box and WD repeat domain‐containing 7
FBXO42
: F‐box only protein 42
GABARAP
: Gamma‐aminobutyric acid receptor‐associated protein
GABARAPL1/2
: GABARAP like 1/2
GADD34
: Growth arrest and DNA damage‐inducible protein 34
GATE16
: Golgi‐associated ATPase enhancer of 16 KDa
GCN2
: General control nonderepressible 2
GRINA
: Glutamate ionotropic receptor N‐methyl‐D‐aspartate type subunit associated protein
GRP78
: Glucose‐regulated protein, 78 kDa
HERP1/2
: Hairy/enhancer‐of‐split‐related repressor protein 1/2
HERPUD1
: Homocysteine inducible ER protein with ubiquitin like domain 1
HLA‐A2
: HLA class I histocompatibility antigen, A alpha chain 2
HRD1
: Hydroxymethylglutaryl‐CoA reductase degradation 1 homolog
HRI
: Heme‐regulated eIF2α kinase
HSPA5
: Heat shock protein A5
IAP
: Inhibitor of apoptosis protein
INSIG1/2
: Insulin‐induced gene 1/2 protein
IP3R
: Inositol‐1,4,5‐trisphosphate receptor
IRE1
: Inositol‐requiring enzyme 1
ISG15
: Interferon‐stimulated gene 15
JNK
: C‐Jun N‐terminal kinase 1
KLHDC10
: Kelch domain‐containing 10
LC3
: Light chain 3
LTN1
: Listerin E3 ubiquitin protein ligase 1
ManIA
: Mannosidase IA
MAP1LC3B
: Microtubule‐associated protein 1 light chain 3 beta
MARCH5/6
: Membrane‐associated RING‐CH‐type finger 5/6
MCS
: Membrane contact sites
MFN1/2
: Mitofusin 1/2
MITOL
: Mitochodrial ubiquitin ligase
MULAN
: Mitochondrial ubiquitin ligase activator of NFKB
NBR1
: Neighbor of BRCA1 gene 1
NEDD8
: Neural precursor cell expressed, developmentally down‐regulated 8
NEMF
: Nuclear export mediator factor
NOD2
: Nucleotide binding oligomerization domain‐containing 2
NPL4
: Nuclear protein localization 4
OS9
: Osteosarcoma amplified 9
OST
: Oligosaccharyltransferase
OTUB1
: Otubain‐1 deubiquitinase, ubiquitin aldehyde binding 1
PDIA5
: Protein disulfide isomerase A5
PERK
: Protein kinase R‐like endoplasmic reticulum kinase
PHD1/3
: Prolyl hydroxylase domain protein 1/3
PIRH2
: P53‐induced RING‐H2 protein
PKR
: Protein kinase R
PP1/2A
: Protein phosphatase 1/2A
PTM
: Post‐translational modification
PUMA
: P53‐upregulated modulator of apoptosis
QC
: Quality control
RETREG1
: Reticulophagy regulator 1
RHBDL4
: Rhomboid‐like protein 4
RIDD
: Regulated IRE1‐dependent decay of RNAs
RIDDLE
: RIDD‐lacking endomotif
RNF4/5/126/145/170/185/186
: RING finger protein 4/5/126/145/170/185/186
RPL26
: Ribosomal protein L26
RPN1
: Ribophorin I
RPS2/20
: Ribosomal protein S2/20
RQC
: Ribosome‐associated QC
RTCB
: RNA 2″,3″‐cyclic phosphate and 5″‐OH ligase
S1P/S2P
: Site 1/2 proteases
sAIM
: shuffled ATG8‐interacting motif
SAYSD1
: SAYSvFN motif domain‐containing 1
SCF
: S‐phase kinase‐associated protein 1–Cullin1–F–box protein
SEL1L
: Sel‐1 suppressor of Lin‐12‐like 1
SENP3
: SUMO1/Sentrin/SMT3 specific peptidase 3
SGTA
: Small glutamine‐rich tetratricopeptide repeat co‐chaperone alpha
SIAH2
: Seven in absentia homolog 2
SMAC
: Second mitochondria‐derived activator of caspases
SPC
: Signal peptide complex
SPOP
: Speckle type POZ protein
SPP
: Signal peptide peptidase
SUMO
: Small ubiquitin like modifier
SYVN1
: Synovial apoptosis inhibitor 1
TEX264
: Testis expressed 264
TF
: Transcription factor
TMEM129
: Transmembrane protein 129
TNF
: Tumor necrosis factor
TRAF2/6
: TNF receptor‐associated factor 2/6
TRAIL
: TNF‐related apoptosis‐inducing ligand
TRAILR1/2
: TRAIL receptor 1/2
TRAPP
: Trafficking protein particle
TRC35
: Transmembrane domain recognition complex subunit of 35 kDa
TRC8
: Translocation in renal carcinoma on chromosome 8 protein
TXNCD11
: Thioredoxin domain‐containing 11
Ub
: Ubiquitin
UBA5
: Ubiquitin‐like modifier activating enzyme 5
UBAC2
: Ubiquitin‐associated domain‐containing protein 2
UBD
: Ubiquitin‐like modifier D
UBE2J1/J2/D3/2K/2L6
: Ubiquitin conjugating enzyme E2 J1/J2/D3/2K/2L6
Ubl
: Ubiquitin‐like
UBL4A
: Ubiquitin like 4A
UBXD8
: UBX domain‐containing protein 8
UFBP1
: UFM1‐binding protein 1
UFC1
: Ubiquitin‐fold modifier conjugating enzyme 1
UFD1
: Ubiquitin recognition factor In ER‐associated degradation 1
UFIM
: Ubiquitin‐fold modifier 1‐interacting motif
UFL1
: UFM1‐specific ligase 1
UFM1
: Ubiquitin fold modifier 1
UFSP1/2
: UFM1‐specific peptidase 1/2
uL24
: Large ribosomal subunit protein UL24
UPR
: Unfolded protein response
URM1
: Ubiquitin‐related modifier 1
uS3/10
: Small ribosomal subunit protein US4/10
UTR
: Untranslated region
VCP
: Valosin‐containing protein
WSP1
: Wiskott–Aldrich syndrome protein
XBP1s
: X‐box binding protein 1 spliced
XBP1u
: X‐box binding protein 1 unspliced
XTP3B
: XTP3‐transactivated protein B
ZNF598
: Zinc‐finger protein 598

## Introduction

The endoplasmic reticulum (ER) is a central cellular organelle, that plays a key role in calcium homeostasis, lipid metabolism, and protein homeostasis (proteostasis). ER proteostasis is frequently altered, leading to ER stress, in both physiological and pathophysiological conditions. For instance, professional secretory cells (e.g., plasma cells, pancreatic beta‐cells) are constantly subjected to ER stress. Alterations in ER proteostasis are also observed in plethora of pathologies, including cancer, metabolic disorders, or neurodegenerative diseases, driven by intrinsic (e.g., expression of protein variants prone to aggregation, increased secretory demand or oncogene expression), and extrinsic cues (e.g., hypoxia, nutrient deprivation) [1, 2]. To face ER proteotoxicity, cells possess a genetically encoded program called the unfolded protein response (UPR). This response relies on the activation of three ER transmembrane stress sensors, namely PERK, ATF6α, and IRE1α (hereafter referred to as ATF6 and IRE1, respectively). Each of the UPR sensors can be activated upon the accumulation of unfolded or misfolded proteins in the ER lumen. Activation of PERK, IRE1, and ATF6 regulates gene expression at different levels, thereby controlling multiple machineries that contribute to the maintenance of ER proteostasis. These machineries include factors involved in ER pre‐emptive‐quality control (QC), ER protein translocation and folding, ER‐associated degradation (ERAD), ER‐phagy, protein export from the ER as well as ER communication with other organelles through the formation of membrane contact sites (MCS). Indeed, the primary aim of the UPR is to restore ER proteostasis (adaptive UPR). However, a prolonged or intense activation of the UPR can ultimately lead to cell death (terminal UPR) [3, 4]. The transition toward a terminal UPR is often dysregulated in pathological conditions characterized by chronically elevated ER stress, as is the outcome of UPR activation, through dysregulation of the UPR components and/or of the machineries they control. Since the ER stress response can have major effects on cell fate (literally a matter of cell life or death indeed), it is perhaps not surprising that this process is highly regulated by post‐translational modifications (PTMs), particularly ubiquitin (Ub) and ubiquitin‐like (Ubl) modifications, which provide remarkable versatility among PTMs (see below). Here, we first briefly introduce the Ub and Ubl systems. We then focus on selected examples to discuss the different functions of Ub and Ubl, as well as the machineries involved in their metabolism, as mediators and regulators of the ER stress response and its subsequent impact on cell fate.

## Ubiquitin and ubiquitin‐like modifications in a nutshell

Ubiquitin (Ub) and ubiquitin‐like (Ubl) PTMs families play major roles in cellular responses to a wide range of extrinsic and intrinsic stresses [5], including cellular response to DNA damage [6, 7], innate and adaptive immune signaling [8, 9], organelle stress [10], as well as oxidative and temperature stresses [5]. All Ub and Ubl share a structural feature, the β‐grasp fold (for review, see [11]). Ubl can be divided in type I and type II groups. Type I Ubl, including NEDD8, SUMO, ATG8, ATG12, ISG15, URM1, UFM1, and FAT10, are conjugated to substrates in a similar way to Ub. Some of these are actually gathered in families such as the SUMO (including SUMO 1 to 4) and ATG8 (comprising LC3A, LC3B, LC3B2, LC3C, GABARAP, GABARAPL1, and GABARAPL2 (also named GATE16)) families. In contrast, type II Ubl are not conjugated to substrates but instead are part of multidomain proteins that are mostly involved in Ub/Ubl transfer, recognition, or removal. In this review, we will focus on Ub and type I Ubl (hereafter referred to as Ubl), mainly covalently attached to their protein substrates through a primary amine of lysine residues. One notable exception is ATG8, which is conjugated to phosphoethanolamine and, in some cases, to phosphatidylserine [12]. As recently demonstrated for Ub, attachment to non‐proteinaceous substrates may also occur for other Ubl, as well as conjugation to additional amino acid residues (Ser, Thr, Cys, or N‐term Met) [13, 14].

In brief, the covalent attachment of Ub/Ubl to substrates is a three‐step process that involves the ATP‐dependent activation of the Ub/Ubl by an E1 enzyme first, followed by its conjugation by an E2 enzyme, and lastly its transfer to the substrate by an E3 ligase [11]. Ub/Ubl can be attached to substrates as single units (mono‐Ub/Ubl modifications), but may also be extended into chains (poly‐Ub/Ubl modifications) with diverse topologies depending on the residue involved in the inter‐Ub/Ubl linkage formation [15]. Additional PTMs may further modify Ub/Ubl (e.g., phosphorylation, acetylation, etc), thereby expanding the complexity of the Ub/Ubl code [13]. Finally, Ub‐Ubl hybrid chains have also been reported, for example, SUMO‐ubiquitin, NEDD8‐ubiquitin, and NEDD8‐SUMO hybrid chains [16, 17, 18]. Ub/Ubl PTMs are subsequently recognized by specific proteins, referred to as “readers” which display domains able to sense individual Ub/Ubl, different assemblies as Ub/Ubl chains, and/or the modified substrate.

Human Ub and Ubl transfer and removal machineries range from highly complex systems (e.g., the Ub system comprises 2 E1, 40 E2s, more than 600 E3, around 100 deubiquitinases, and likely several hundreds of readers) to seemingly much simpler systems (e.g., the UFM1 system includes 1 E1, 1 E2, 1 E3, 2 deufmylases, and a handful of readers so far [19, 20]). This variation in system complexity likely reflects biological realities. Indeed, some Ubl may be highly specialized to a limited number of substrates and cellular pathways whereas Ub is currently considered a more generalist PTM. This disparity probably also reflects the current limitations of our knowledge on most Ubl systems compared to the Ub one. Hereafter, we will highlight the multiple roles played by Ub and Ubl modifications in the cellular response to ER stress, using examples on three selected processes, and focusing on mammalian cells unless otherwise specified.

## Ub and Ubl functions in the UPR: Responding to unfolded/misfolded protein accumulation in the ER

**Fig. 1:**
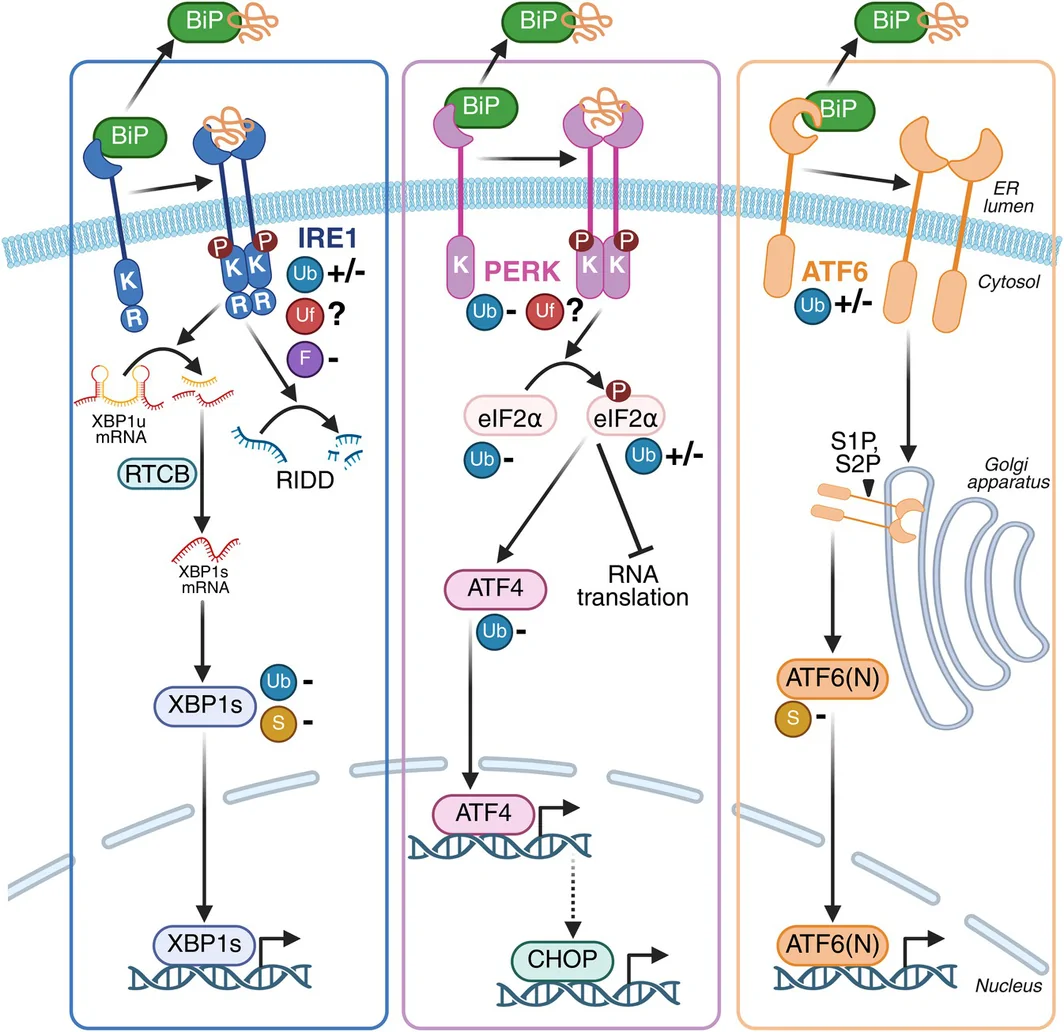
The unfolded protein response. Upon accumulation of misfolded proteins in the ER lumen, three sensors, namely IRE1, PERK and ATF6, can be activated leading to modulation of gene expression through distinct mechanisms. Ubiquitin (Ub) modification can positively or negatively regulate each of these branches at different steps (see main text). SUMO (S) modification negatively regulates ATF6 and XBP1s, thereby repressing their transcriptional activity. UFM1 (Uf) modification can also regulate the IRE1 and PERK branches, although the exact mechanisms remain unclear. Of note, FAT10 (F) interacts with IRE1 in a non‐covalent manner, while its impact on RNase activity remains unknown. ATF4/6, Activating transcription factor 4/6; ATF6(N), N‐terminal fragment of ATF6; BiP, Binding‐immunoglobulin protein; eIF2α, Eukaryotic translation initiation factor 2 alpha; ER, Endoplasmic reticulum; FAT10, HLA‐F adjacent transcript 10; IRE1, Inositol‐requiring enzyme 1; K, Kinase domain; P, Phosphorylation; PERK, Protein kinase R‐like endoplasmic reticulum kinase; R, RNase domain; RIDD, Regulated IRE1‐dependent decay of RNAs; RTCB, RNA 2′,3′‐cyclic phosphate and 5′‐OH ligase; SUMO, Small ubiquitin like modifier; S1P/S2P, Site 1/2 proteases; UFM1, Ubiquitin fold modifier 1; XBP1s, X‐box binding protein 1 spliced; XBP1u, X‐box binding protein 1 unspliced.

Upon accumulation of un/misfolded proteins in the ER lumen, the sensors ATF6, PERK, and IRE1 are activated, driving the UPR [21, 22] (Fig. 1) whose primary role is to restore ER proteostasis. Notably, disruption of ER lipid homeostasis can also lead to activation of UPR sensors (e.g., activation of ATF6 by dihydrosphingosine and dihydroceramide [23]). Increasing evidence now emerges on the involvement of Ub and Ubl in the UPR. As detailed below, this includes (i) modulation of stability and/or activation of ATF6, PERK, IRE1 and downstream effectors by Ub and Ubl as well as (ii) regulation of components of the Ub and Ubl machineries downstream of the different UPR branches to control ER proteostasis.

ATF6 is a type II transmembrane ER protein, residing as monomers and interchain disulfide‐stabilized dimers [24]. Upon ER stress, ATF6 activation involves the release of the chaperone BiP (also known as HSPA5, or GRP78) from its luminal domain, as BiP preferentially binds mis/unfolded proteins. This release exposes ATF6 Golgi localization signals [25] as well as intra‐monomer disulfide bonds [24].

The protein disulfide isomerases (PDI) PDIA5 and ERP18 [26, 27, 28] facilitate ATF6 dimer formation which promotes preferentially its export to the Golgi apparatus through COP‐II vesicles [25, 29]. In the Golgi, ATF6 is sequentially cleaved by site 1 and site 2 proteases (S1P and S2P), releasing a cytosolic N‐terminal fragment, ATF6(N), which translocates to the nucleus and functions as a transcription factor to induce genes involved in restoring ER homeostasis [30, 31]. These target genes include components of the ERAD machinery, factors involved in protein folding and secretion, ER and Golgi biogenesis, as well as XBP1u (see below) [32, 33, 34, 35, 36].

Recent evidence indicates a role for Ub modification in the ATF6 pathway. Several studies have shown that Ub controls ATF6 stability and/or activation. Under basal conditions, ATF6 is relatively unstable, with a reported half‐life of around 2 h in human cells [31], and degraded through ERAD, a Ub‐dependent process (see below). Upon thapsigargin‐induced ER stress, full‐length ATF6 undergoes an early and transient proteasome‐dependent degradation that is independent of its cleavage by S1P/S2P [37]. Mechanistically, ERAD‐mediated degradation of ATF6 requires HRD1, SEL1L, DERLIN2/3, HERP1/2 as well as trimming of mannose, involving EDEM1 [38, 39, 40]. Both basal and ER stress‐induced ATF6 degradation likely contribute to attenuate its activation during ER stress [39]. HRD1‐dependent ATF6 degradation is also modulated by various HRD1 or ATF6 interactors, as illustrated by GRINA in the context of hepatic ischemia–reperfusion [41]; and by WSP1 [42], a protein whose mutation causes Wolfram syndrome, a rare neurodegenerative disorder characterized by multiple defects of ER proteostasis regulation, including impaired ER‐mitochondria contact sites formation and function [43]. Additional extrinsic cues may further modulate ATF6 activation by altering its ubiquitination status. For instance, engagement of the pattern recognition receptor NOD2 in macrophages promotes ATF6 ubiquitination by the E3 Ub ligase RNF186, that promotes its activation, ensuing cytokine production and bacterial clearance [44], although the underlying molecular mechanisms remain to be clarified. Similarly, glucose exposure of skeletal muscle cells and hepatocytes induces RNF186 expression, thereby contributing to ATF6 stabilization and activation [45]. ATF6 can also be modified by SUMOylation, resulting in repression of its transcriptional activity [46]. Whether additional Ubl directly regulate ATF6 stability or activation remains an open question.

Once translocated to the nucleus, ATF6(N) induces the expression of numerous genes, including several components of the Ub/Ubl system involved in ER proteostasis. For example, ATF6(N) upregulates multiple ERAD components, including HERPUD1, SEL1L, DERLIN3, and EDEM1 [33, 47, 48]. ATF6(N) can also indirectly promote expression of the ERAD E3 ligase HRD1 (also named SYVN1) [33, 49], whereas its transcription is directly induced by the IRE1/XBP1s branch ([50, 51], see below). ATF6(N) also contributes to the expression of some Ubl themselves. For instance, the transcription factor CHOP, acting downstream of ATF6 and PERK, induces the expression of LC3B [52]. Altogether, these examples position Ub and Ubl PTMs as both key regulators and mediators of ATF6 activation during ER stress response.

PERK (also known as EIF2AK3) is a type I ER transmembrane protein. As ATF6, its activation involves detachment of its luminal domain from the chaperone BiP, leading to PERK dimerization, activation of its cytosolic kinase domain and auto‐phosphorylation [1, 2, 21]. Activated PERK phosphorylates eIF2 alpha (eIF2α), thereby inhibiting translation initiation, and reducing overall protein synthesis [53]. Despite this global translational repression, a subset of mRNAs are still translated due to the presence of one or more upstream open reading frames in their 5′UTR, including the one encoding for the transcription factor ATF4 [54, 55, 56]. ATF4, in turn, initiates the transcription of genes involved in restoration of ER proteostasis, including autophagy, amino acid metabolism, ER protein folding, and redox response. Depending on cellular context, ATF4 also contributes to the terminal UPR, often in collaboration with CHOP, by inducing the expression of pro‐apoptotic BCL‐2 family members (e.g., BIM, NOXA, PUMA, BAX, BAK, BCL‐2), death receptors TRAIL‐R1/2 and their ligand TRAIL [4, 57]. In addition to its broader transcriptional program, CHOP induces the expression of ER oxidoreductin 1α (ERO1α) [58]. ERO1α reoxidizes protein disulfide isomerases (PDIs), thereby promoting oxidative protein folding of ER client proteins, a process that is intrinsically coupled to hydrogen peroxide (H₂O₂) generation and normally buffered by ER‐localized peroxidases. Under conditions of severe or prolonged ER stress, however, CHOP‐driven upregulation of ERO1α can exacerbate oxidative stress and contribute to apoptosis [59]. Mechanistically, increased ERO1α activity promotes hyperoxidation of the ER lumen and enhances inositol 1,4,5‐trisphosphate receptor (IP₃R)‐mediated Ca^2+^ release, thereby linking ER redox imbalance to calcium‐dependent apoptotic signaling. Of note, eIF2α phosphorylation is also mediated by three other kinases, HRI (upon mitochondrial alteration or heme depletion), PKR (in viral infection), and GCN2 (upon amino acid depletion). This phosphorylation is reversible through the activity of phosphatases complexes GADD34/PP1 and CREP/PP1. Notably, GADD34 is CHOP‐inducible, providing a negative feedback loop to restore translation [58, 60, 61]. Together with PERK, HRI, PKR, and GCN2 kinases reprogram translation and mediate the integrated stress response (ISR) [62]. Like the UPR, the ISR primarily promotes cellular adaptation to stress but can also drive cell death under conditions of prolonged stress and is dysregulated in various diseases.

Several studies now highlight an Ub‐dependent regulation of the PERK pathway at different steps. First, PERK stability and activation are regulated by ubiquitination. Indeed, the BRCA1‐BARD1 complex, in association with the ERAD components DERLIN1 and SEL1L, ubiquitinates and limits PERK and IRE1 stability [63]. In cancer cells with BRCA1 loss‐of‐function, PERK and IRE1 are more stable, leading to enhanced UPR activation that helps to manage the increased load of misfolded proteins and ensures tumor cell survival. Disrupting this adaptation may thus represent an attractive therapeutic target for BRCA1‐mutated cancer. Interestingly, deletion of UFBP1 (also known as DDRGK1), a core component of the UFMylation machinery, increases PERK expression and activation in murine plasma cells, although the exact mechanism remains unclear and may be indirect. UFBP1 deletion also impairs plasma cell development in a PERK‐dependent manner [64]. Downstream PERK pathway components are also regulated by Ub. Indeed, eIF2α and phosphorylated eIF2α are ubiquitinated and targeted for degradation after HDR1 overexpression [65]. In contrast, RNF4‐mediated ubiquitination of phosphorylated eIF2α induces its stabilization in melanoma cells [66]. In addition, SCF‐bTrCP ubiquitinates ATF4, promoting its degradation [67, 68], while both E3 Ub ligases PARKIN and cIAP1 destabilize CHOP, contributing to resistance against ER stress‐induced cell death in cardiomyocytes and β‐cells respectively [69, 70].

Conversely, PERK modulates the expression and activity of several Ub/Ubl machinery components directly through its kinase activity or via ATF4/CHOP. Indeed, PERK phosphorylates the Ub E3 ligases PARKIN, MARCH5 and MULAN, increasing their activity toward ER‐mitochondria‐associated targets and therefore reducing ER‐mitochondria contact sites formation and mitochondrial energy production [71]. UBE2J1, a Ub E2 important in ERAD, is phosphorylated downstream of PERK during ER stress [72], improving ER stress recovery [73]. PERK also enhances the expression of the E3 ligases cIAP1/2, through both translational and transcriptional regulations, repressing cell death induction upon ER stress [74]. Consistently, pharmacological inhibition of IAPs (e.g., by SMAC mimetics) has been explored to sensitize cancer cells to ER stress‐induced cell death (among other types of stress‐induced death). Additional PERK‐dependent effects include ubiquitination of the 40S ribosomal proteins RPS2 and RPS20, which contributes to repressed cell death upon ER stress [75]. ATF4 also promotes the expression of several ubiquitin ligases which play a role in ER stress adaptation. For example, PARKIN, an ATF4 target, contributes to repressing ER stress‐induced cell death by limiting mitochondrial damage [76]. SIAH2, induced by both ATF4 and XBP1s, inactivates PHD1/3, thereby stabilizing ATF4 by limiting proline hydroxylation [77]. Regarding Ubl, PERK activation induces ATG8 and ATG12 upon ER stress [78]. In human prostate adenocarcinoma cells LNCaP, ATF4 (independently of PERK) induces LC3A, LC3B, LC3B2, LC3C and GABARAPL1 expression, facilitating autophagosome formation; while PERK (independently of ATF4) impacts autophagy at a later step of ER stress [79]. In MEFs, PERK promotes expression of multiple autophagy genes (including ATG16L1, MAP1LC3B, ATG12, ATG3, BECLIN1, GABARAPL2, p62, NBR1, ATG7, ATG10, GABARAP, and ATG5) via ATF4 and/or CHOP [80]. LC3B expression is also induced by ATF4 upon hypoxia, promoting autophagosome formation and cell survival [81]. During hepatitis C virus infection, the PERK/ATF4 axis and CHOP promote ATG12 and LC3B, respectively [52]. PERK additionally promotes ATG8, along ATG5, ATG7 and BECLIN1 expression, in response to extracellular matrix detachment, promoting cell survival [82]. ATF4 activation further contributes to autophagic cell death and reticulophagy, by inducing the LC3/GABARAP‐binding reticulophagy receptors RETREG1/FAM134B and, to a lesser extent, TEX264 [83]. Collectively, these findings highlight that PERK, ATF4 and/or CHOP orchestrate several steps of autophagy in various cellular stress contexts, with key consequences on cell adaptation to stress. Finally, during ischaemia, PERK‐ and CTSB‐dependent degradation of the SUMO‐specific protease SENP3 increases global SUMO2/3 conjugation, including of DRP1, limiting its mitochondrial association, cytochrome c release and cell death. Upon reoxygenation, SENP3 levels rise again, promoting cell death [84].

IRE1 activation occurs following detachment of its luminal domain from BiP and/or direct binding to misfolded proteins. This triggers its oligomerisation, activation of its cytosolic kinase domain, and subsequent activation of its cytosolic RNase domain. Indeed, IRE1 auto‐phosphorylates which promotes its RNase activity; and its kinase activity also targets other substrates that can regulate its RNase function (e.g., PUMILLIO [85]). The RNase activity of IRE1 results in two main outcomes. First, IRE1 cleaves XBP1u (for unspliced) mRNA at two harpin loops, removing a 26‐nucleotide intron. The resulting fragments are ligated by the tRNA ligase RTCB, generating the XBP1s mRNA, which is translated to the XBP1s transcription factor. XBP1s induces expression of multiple genes mainly involved in ER proteostasis, such as chaperones, PDIs and ERAD components. Intriguingly, recent evidence suggests that XBP1s can also act as a transcriptional repressor [86]. Second, the IRE1 RNase degrades various mRNA and non‐coding RNAs, through the regulated IRE1‐dependent decay of RNAs (RIDD), which relies on specific RNA structures/sequences on targeted RNAs [87], and directly contributes to reducing ER protein load. Notably, RIDD targets are not limited to mRNA encoding ER‐resident or secretory proteins; they are in fact, involved in a large variety of processes including inflammation, cell migration, cell death, lipid metabolism, lysosomal trafficking, as well as circadian rhythm [87]. While the XBP1s branch is generally viewed as adaptative, the RIDD branch (often triggered by prolonged ER stress) contributes to both adaptive and terminal UPR. Some targets, we recently termed dual IRE1 targets [88, 89], are induced downstream of XBP1s, and repressed by RIDD activity, likely providing their fine‐tuning regulation upon response to stress [90]. Additionally, a more promiscuous RNA degradation activity, termed RIDDLE (for RIDD‐lacking endomotif), has been described, though its biological functions and trigger circumstances remain under investigation [91]. Finally, IRE1 can activate inflammatory and apoptotic pathways through a TRAF2/ASK1/JNK‐mediated mechanism, illustrating its function as a scaffold for cell signaling beyond RNase activity, as recently reviewed in [92].

Among the UPR branches, IRE1 is the best‐characterized for Ub/Ubl involvement. IRE1 is ubiquitinated by multiple E3 ligases that control its stability and activation. For instance, the ERAD E3 ligase HRD1 regulates IRE1 stability [93, 94]; similarly, BRCA1 also ubiquitinates and destabilizes IRE1 [63]. K63‐ubiquitination of IRE1 by CHIP on K545 and K828, respectively, enhances its phosphorylation and TRAF2 interaction, promoting JNK and p38 activation upon ER stress [95]. This modification is repressed by the deubiquitinase OTUB1. The effect of CHIP‐mediated ubiquitination on IRE1 RNase activity remains to be evaluated. Interestingly, IRE1 function is regulated by MCS formation as the mitochondrial E3 ligase MITOL ubiquitinates IRE1 on K481 with K63‐linked chains upon ER stress, limiting its hyper‐oligomerization, thereby repressing its RIDD activity and apoptosis [96]. Upon LPS stimulation, TRAF6 ubiquitinates IRE1, repressing its interaction with the phosphatase PP2A and thereby promoting IRE1 activation [97]. IRE1 stability and activation are also regulated by UFMylation [19, 20, 98, 99], although conflicting results are reported [64, 100, 101, 102, 103]. Intriguingly, UBD/FAT10 interacts non‐covalently with IRE1, limiting JNK activation [104].

Downstream of IRE1, XBP1 is also subjected to Ub/Ubl modifications. For example, XBP1u is an unstable protein with a half‐life of 11 min [105] (as compared to an average half‐life of several hours for human proteins [106, 107]), a feature that involves both ubiquitin‐independent and ‐dependent processes [108, 109, 110]. XBP1s is also unstable (22 min half‐life, [105]) and its ubiquitination at K60 and K77 promotes its proteasome‐mediated degradation [111]. Several Ub E3 ligases have been proposed to repress XBP1s stability, including CULLIN3‐SPOP [112], SKP1‐CULLIN1‐FBW7 [113]. XBP1s may also be SUMOylated, a process which represses its transcriptional activity [114]. Intriguingly, XBP1 translation can also be repressed through its isolation in ATAXIN2 granules, a phenomenon that is counteracted upon ER stress by FBXO42 and ubiquitin‐dependent degradation of ATAXIN2, promoting XBP1s expression and cell death induction [115]. Importantly, XBP1s targets include many components of the Ub/Ubl systems, notably the UFMylation and deUFMylation machineries (UFM1, UBA5, UFC1, UFL1, CDK5RAP3, DDRGK1, UFSP1, UFSP2) [19, 20, 98, 99]. Several ERAD components, including the E3 ligase HRD1, SEL1L, HERPUD1, and EDEM1, are also directly induced by XBP1s [47, 48, 49, 51]. IRE1 kinase domain also controls Ub and Ubl‐dependent processes; for instance, IRE1 deficiency limits LC3‐dots formation upon ER stress [116].

**Fig. 2:**
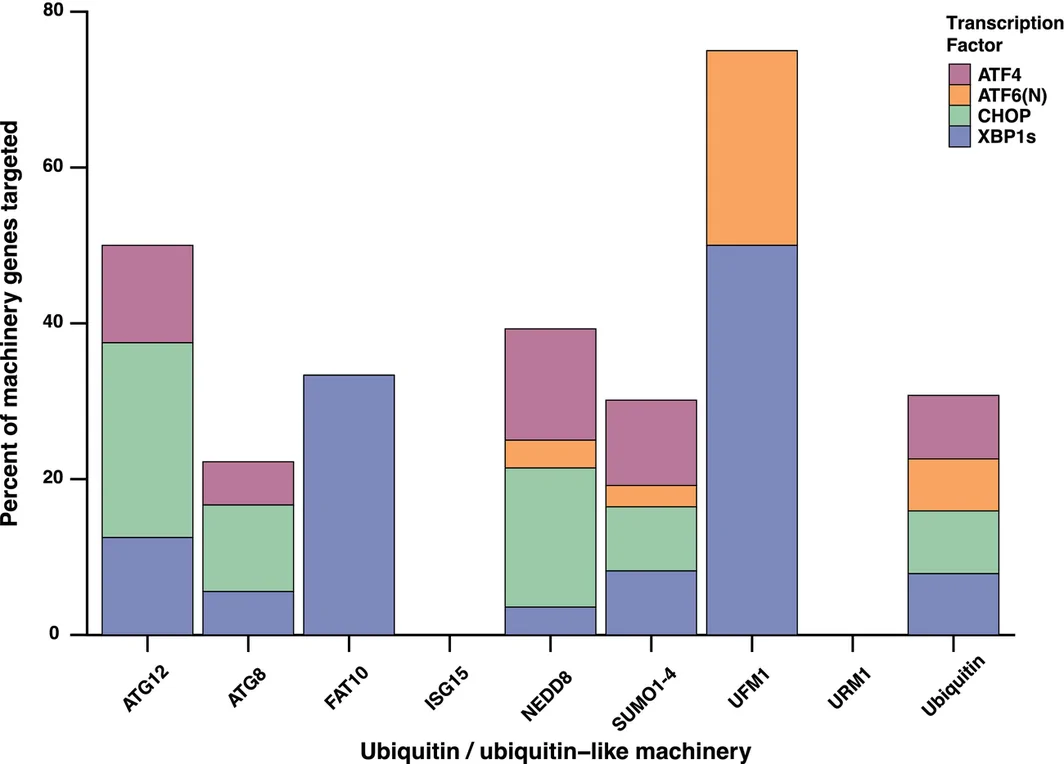
Unfolded protein response transcription factor coverage of ubiquitin and ubiquitin‐like machineries. Stacked bar plot showing the percentage of genes belonging to each ubiquitin‐like functional machinery, as well as the ubiquitin machinery, that are targeted by individual UPR TFs. For each machinery, gene coverage was calculated as the proportion of annotated genes present among the top 1000 ChIP‐Atlas targets of each TF. Genes annotated to multiple ubiquitin‐like machineries were counted independently in each corresponding category. Genes shared between ubiquitin and ubiquitin‐like annotations were assigned exclusively to the ubiquitin‐like category. Colors denote individual TFs. See also methods and Tables 1 and 2. ATF4/6, Activating transcription factor 4/6; ATF6(N), N‐terminal fragment of ATF6; ATG8/12, Autophagy‐related 8/12; ChIP, Chromatin immunoprecipitation; FAT10, HLA‐F adjacent transcript 10; ISG15, Interferon‐stimulated gene 15; NEDD8, Neural precursor cell expressed developmentally down‐regulated 8; SUMO 1–4, Small ubiquitin like modifier isoforms 1 to 4; TF, Transcription factor; UFM1, Ubiquitin fold modifier 1; UPR, Unfolded protein response; URM1, Ubiquitin related modifier 1.

Overall, these findings illustrate that Ub/Ubl modifications both mediate and regulate UPR. Considering their reported target sites (Fig. 2, Tables 1 and 2), TFs from all UPR branches are likely to regulate the Ub machinery. Notably, XBP1s appears to regulate most of the Ubl machineries. In contrast, ATF6 may be more specialized in controlling SUMO, UFM1 and NEDD8 machineries, which could indicate a preferential role in controlling the crosstalk between DNA damage response and ER stress response (via SUMOylation) and ubiquitin regulation, through NEDDylation as well as fine‐tuning ER proteostasis through UFMylation. Similarly, ATF4 and CHOP may preferentially control autophagy through ATGylation as well as fine‐tuning ubiquitination via NEDD8, and DNA damage response through SUMOylation. Beyond their individual roles, evidence for crosstalk between UPR branches in the control of ER proteostasis has emerged (e.g., [117, 118, 119]). Analogous to phosphorylation events [119], and considering that many Ub/Ubl machinery components are regulated by each UPR branch, it is highly likely that Ub/Ubl modifications are also involved in these crosstalks.

**Table 1:** Ubiquitin‐like‐associated genes and corresponding GO terms.

SYMBOL	Machinery	GO_terms
ABCE1	UFM1	GO:0071569
ASCC3	UFM1	GO:0071569
ATG10	ATG12	GO:0019777;GO:0061651
ATG12	ATG12;ATG8	GO:0019776;GO:0034274
ATG16L1	ATG12	GO:0034274
ATG16L2	ATG12	GO:0034274
ATG3	ATG12;ATG8	GO:0019776;GO:0019777;GO:0034274;GO:0141046
ATG4A	ATG8	GO:0019786
ATG4B	ATG8	GO:0019786
ATG4C	ATG8	GO:0019786
ATG4D	ATG8	GO:0019786
ATG5	ATG12;ATG8	GO:0019776;GO:0034274
ATG7	ATG12;ATG8	GO:0019778;GO:0019779
ATG8	ATG8	NA
BCL11A	SUMO1_4	GO:0016925
CASP8AP2	SUMO1_4	GO:0032184
CBX4	SUMO1_4	GO:0016925;GO:0019789;GO:0032183
CDK5RAP3	UFM1	GO:0071569
CDKN2A	SUMO1_4	GO:0016925;GO:0019789
COPS2	NEDD8	GO:0000338;GO:0045116
COPS3	NEDD8	GO:0000338;GO:0045116
COPS4	NEDD8	GO:0000338;GO:0019784;GO:0045116
COPS5	NEDD8	GO:0000338;GO:0019784;GO:0045116
COPS6	NEDD8	GO:0000338;GO:0045116
COPS7A	NEDD8	GO:0000338;GO:0045116
COPS7B	NEDD8	GO:0000338
COPS8	NEDD8	GO:0000338;GO:0045116
CTU1	URM1	GO:0032447
CTU2	URM1	GO:0032447
DCUN1D1	NEDD8;URM1	GO:0045116
DCUN1D2	NEDD8	GO:0045116
DCUN1D3	NEDD8	GO:0045116
DCUN1D4	NEDD8	GO:0045116
DCUN1D5	NEDD8	GO:0045116
DDRGK1	UFM1	GO:0071569;GO:0141185;GO:1990592
DESI1	SUMO1_4	GO:0016926;GO:0016929
EGR2	SUMO1_4	GO:0016925
EID3	SUMO1_4	GO:0016925
EYA1	SUMO1_4	GO:0016925
GABARAP	ATG8	NA
GABARAPL1	ATG8	NA
GABARAPL2	ATG8	NA
GCNA	SUMO1_4	GO:0032184
GPS1	NEDD8	GO:0000338;GO:0045116
HABP4	SUMO1_4	GO:0032183
HDAC4	SUMO1_4	GO:0016925;GO:0019789
HDAC7	SUMO1_4	GO:0016925;GO:0019789
HERC2	SUMO1_4	GO:0032183
HERC5	ISG15	GO:0032020;GO:0042296
HINT1	SUMO1_4	GO:0016926;GO:0016929
HMG20B	SUMO1_4	GO:0016925
IFIH1	SUMO1_4	GO:0016925
ISG15	ISG15	GO:0032020
KIAA1586	SUMO1_4	GO:0016925
LC3A	ATG8	NA
LC3B	ATG8	NA
LC3B2	ATG8	NA
LC3C	ATG8	NA
MDM2	NEDD8;SUMO1_4	GO:0016925;GO:0019789;GO:0061663
MOCS3	URM1	GO:0032447;GO:0042292
MUL1	SUMO1_4	GO:0016925;GO:0019789
NAE1	NEDD8;URM1	GO:0019781;GO:0045116
NEDD8	NEDD8	GO:0045116
NFATC2IP	SUMO1_4	GO:0016925
NSMCE1	SUMO1_4	GO:0016925
NSMCE2	SUMO1_4	GO:0016925;GO:0019789
NSMCE3	SUMO1_4	GO:0016925
NSMCE4A	SUMO1_4	GO:0016925
OTUB1	NEDD8	GO:0019784
PELP1	SUMO1_4	GO:0032183
PIAS1	SUMO1_4	GO:0016925;GO:0019789
PIAS2	SUMO1_4	GO:0016925;GO:0019789
PIAS3	SUMO1_4	GO:0016925;GO:0019789
PIAS4	SUMO1_4	GO:0016925;GO:0019789
PML	SUMO1_4	GO:0016925;GO:0019789;GO:0032183
RANBP2	SUMO1_4	GO:0016925;GO:0019789;GO:0106068
RANGAP1	SUMO1_4	GO:0016925;GO:0106068
RBX1	NEDD8	GO:0019788;GO:0045116;GO:0061663
RNF111	SUMO1_4	GO:0032184
RNF212	SUMO1_4	GO:0016925;GO:0019789
RNF212B	SUMO1_4	GO:0019789
RNF4	SUMO1_4	GO:0032184
RNF7	NEDD8	GO:0019788;GO:0045116;GO:0061663
SAE1	SUMO1_4	GO:0016925;GO:0019948;GO:0031510
SAYSD1	UFM1	GO:0141185
SENP1	SUMO1_4	GO:0016925;GO:0016926;GO:0016929;GO:0070139
SENP2	SUMO1_4	GO:0016925;GO:0016926;GO:0016929;GO:0070139
SENP3	SUMO1_4	GO:0016926;GO:0016929
SENP5	SUMO1_4	GO:0016925;GO:0016926;GO:0016929;GO:0070139
SENP6	SUMO1_4	GO:0016925;GO:0016926;GO:0070139
SENP7	SUMO1_4	GO:0016926;GO:0070139
SENP8	NEDD8	GO:0000338;GO:0019784
SERBP1	SUMO1_4	GO:0032183
SIMC1	SUMO1_4	GO:0032184
SLF1	SUMO1_4	GO:0016925
SLF2	SUMO1_4	GO:0016925
SMC5	SUMO1_4	GO:0016925
SMC6	SUMO1_4	GO:0016925
SOBP	SUMO1_4	GO:0032184
STK38	UFM1	GO:0141185
STX1A	SUMO1_4	GO:0016925
SUMO1	SUMO1_4	GO:0016925
SUMO1P1	SUMO1_4	GO:0016925
SUMO2	SUMO1_4	GO:0016925;GO:0019789
SUMO3	SUMO1_4	GO:0016925
SUMO4	SUMO1_4	GO:0016925;GO:0106068
TDG	SUMO1_4	GO:0032183
TOLLIP	SUMO1_4	GO:0032183
TOPORS	SUMO1_4	GO:0016925;GO:0019789
TOR1A	NEDD8	GO:0000338
TRIM27	SUMO1_4	GO:0016925;GO:0019789
TRIM28	SUMO1_4	GO:0016925;GO:0019789
TRIM38	SUMO1_4	GO:0016925
TRIM40	NEDD8	GO:0045116
TRIM60	SUMO1_4	GO:0140082
TRPM4	SUMO1_4	GO:0016925
UBA2	SUMO1_4	GO:0016925;GO:0019948;GO:0031510;GO:0032183
UBA3	NEDD8;URM1	GO:0019781;GO:0019788;GO:0045116
UBA5	UFM1	GO:0071566;GO:0071569;GO:1990592
UBA6	FAT10	GO:0019780
UBA7	ISG15	GO:0019782;GO:0032020
UBD	ATG8;FAT10	NA
UBE2E1	ISG15	GO:0032020;GO:0042296
UBE2E2	ISG15	GO:0032020;GO:0042296
UBE2F	NEDD8	GO:0019788;GO:0045116;GO:0061654;GO:0061663
UBE2I	SUMO1_4	GO:0016925;GO:0019789;GO:0106068
UBE2L6	ISG15	GO:0032020;GO:0042296
UBE2M	NEDD8;URM1	GO:0019788;GO:0045116;GO:0061654
UBE2Z	ATG8;FAT10	NA
UCHL3	NEDD8	GO:0019784
UFC1	UFM1	GO:0061657;GO:0071568;GO:0071569;GO:1990592
UFL1	UFM1	GO:0061666;GO:0071568;GO:0071569;GO:1990592
UFM1	UFM1	GO:0071569;GO:1990592
UFSP1	UFM1	GO:0071567;GO:0071569
UFSP2	UFM1	GO:0071567
UHRF2	SUMO1_4	GO:0016925;GO:0019789
URM1	URM1	GO:0032447
USP18	ISG15	GO:0019785
USP21	NEDD8	GO:0019784
USP25	SUMO1_4	GO:0032183
USP43	ISG15	GO:0019785
USPL1	SUMO1_4	GO:0016926;GO:0016929;GO:0032183
WDFY3	ATG12	GO:0034274
ZBED1	SUMO1_4	GO:0016925;GO:0019789
ZMIZ1	SUMO1_4	GO:0016925
ZMIZ2	SUMO1_4	GO:0016925
ZNF451	SUMO1_4	GO:0016925

**Table 2:** Ubiquitination‐associated genes and corresponding GO terms.

Gene name	Machinery	GO terms
ABCB1	Ubiquitin	GO:0031625
ABI2	Ubiquitin	GO:0031625
ABRAXAS1	Ubiquitin	GO:0031593
ABRAXAS2	Ubiquitin	GO:0031593
ACTG1	Ubiquitin	GO:0031625
ACVR1B	Ubiquitin	GO:0031625
AFG2B	Ubiquitin	GO:0031593
AGAP3	Ubiquitin	GO:0031593
AGL	Ubiquitin	GO:0031593
AICDA	Ubiquitin	GO:0031625
AKTIP	Ubiquitin	GO:0061631
ALG13	Ubiquitin	GO:0004843
AMBRA1	Ubiquitin	GO:0031625;GO:1990756
AMFR	Ubiquitin	GO:0004842;GO:0034450;GO:0043130;GO:0061630;GO:1990381
ANAPC11	Ubiquitin	GO:0004842;GO:0034450;GO:0061630;GO:0097602
ANAPC2	Ubiquitin	GO:0031625
ANAPC4	Ubiquitin	GO:0004842
ANKIB1	Ubiquitin	GO:0004842;GO:0031624;GO:0061630
ANKRA2	Ubiquitin	GO:0031625
ANKRD13A	Ubiquitin	GO:0140036
ANKRD13B	Ubiquitin	GO:0140036
ANKRD13D	Ubiquitin	GO:0140036
ANKRD9	Ubiquitin	GO:1990756
APBB1	Ubiquitin	GO:0031625
APC	Ubiquitin	GO:0031625
APPBP2	Ubiquitin	GO:1990756
AREL1	Ubiquitin	GO:0004842;GO:0061630
ARHGAP5‐AS1	Ubiquitin	GO:1990948
ARIH1	Ubiquitin	GO:0004842;GO:0019787;GO:0031624;GO:0031625;GO:0061630
ARIH2	Ubiquitin	GO:0004842;GO:0031624;GO:0061630
ARK2C	Ubiquitin	GO:0061630
ARK2N	Ubiquitin	GO:0061630
ARMC5	Ubiquitin	GO:1990756
ARRB1	Ubiquitin	GO:0031625
ARRB2	Ubiquitin	GO:0031625
ARRDC1	Ubiquitin	GO:0031625;GO:1990756
ARRDC4	Ubiquitin	GO:1990756
ASB1	Ubiquitin	GO:0061630;GO:1990756
ASB11	Ubiquitin	GO:1990756
ASB12	Ubiquitin	GO:0061630
ASB2	Ubiquitin	GO:0061630;GO:0097602
ASB4	Ubiquitin	GO:0004842;GO:0031625;GO:0061630
ASB9	Ubiquitin	GO:1990756
ASCC2	Ubiquitin	GO:0043130;GO:0070530
ATP6V0C	Ubiquitin	GO:0031625
ATRIP	Ubiquitin	GO:0070530
ATXN3	Ubiquitin	GO:0004843;GO:0031625;GO:0061578;GO:1990380
ATXN3L	Ubiquitin	GO:0004843
AUP1	Ubiquitin	GO:0031624;GO:0031625;GO:0043130
AURKA	Ubiquitin	GO:0031625
AXIN1	Ubiquitin	GO:0031625;GO:1990756
AXIN2	Ubiquitin	GO:0031625
BABAM2	Ubiquitin	GO:0031593
BAG1	Ubiquitin	GO:0031625
BAG2	Ubiquitin	GO:0031625
BAG4	Ubiquitin	GO:0031625
BAG5	Ubiquitin	GO:0031625;GO:1990948
BAG6	Ubiquitin	GO:0031593;GO:0031625;GO:1990381
BAP1	Ubiquitin	GO:0004843;GO:0140950
BARD1	Ubiquitin	GO:0004842;GO:0061630;GO:0061649;GO:0140863;GO:0140864
BCL10	Ubiquitin	GO:0031625
BCL2	Ubiquitin	GO:0031625
BCOR	Ubiquitin	GO:0004842
BECN1	Ubiquitin	GO:0031625
BFAR	Ubiquitin	GO:0061630
BID	Ubiquitin	GO:0031625
BIRC2	Ubiquitin	GO:0004842;GO:0043130;GO:0061630
BIRC3	Ubiquitin	GO:0004842;GO:0061630
BIRC6	Ubiquitin	GO:0004842;GO:0061630;GO:0061631
BIRC7	Ubiquitin	GO:0004842;GO:0061630
BIRC8	Ubiquitin	GO:0004842;GO:0061630
BLZF1	Ubiquitin	GO:0031625
BMI1	Ubiquitin	GO:0097027
BOK	Ubiquitin	GO:0031625
BRAP	Ubiquitin	GO:0004842;GO:0061630
BRCA1	Ubiquitin	GO:0004842;GO:0031625;GO:0061630;GO:0061649;GO:0140863;GO:0140864
BRCC3	Ubiquitin	GO:0004843;GO:0031593;GO:0061578;GO:0140492
BSPRY	Ubiquitin	GO:0061630
BTBD1	Ubiquitin	GO:0097602
BTRC	Ubiquitin	GO:0061630;GO:1990756;GO:1990757
BUB3	Ubiquitin	GO:0043130
C10orf90	Ubiquitin	GO:0061630
CACUL1	Ubiquitin	GO:0031625
CACYBP	Ubiquitin	GO:0031625
CALR	Ubiquitin	GO:0031625
CAMLG	Ubiquitin	GO:0031625
CASC3	Ubiquitin	GO:0031625
CASP10	Ubiquitin	GO:0031625
CASP8	Ubiquitin	GO:0031625
CBL	Ubiquitin	GO:0004842;GO:0061630
CBLB	Ubiquitin	GO:0004842;GO:0061630
CBLC	Ubiquitin	GO:0004842;GO:0061630
CBLL1	Ubiquitin	GO:0004842;GO:0061630
CBLL2	Ubiquitin	GO:0061630
CBS	Ubiquitin	GO:0031625
CBX8	Ubiquitin	GO:0097027
CCAR1	Ubiquitin	GO:0061630
CCDC22	Ubiquitin	GO:0097602
CCDC38	Ubiquitin	GO:0061649
CCDC50	Ubiquitin	GO:0031625
CCIN	Ubiquitin	GO:1990756
CCNB1	Ubiquitin	GO:0044389
CCNB1IP1	Ubiquitin	GO:0061630
CCNF	Ubiquitin	GO:0010997
CCT2	Ubiquitin	GO:0031625
CD40	Ubiquitin	GO:0031625
CDC20	Ubiquitin	GO:0010997;GO:0097027;GO:1990756;GO:1990757
CDC20B	Ubiquitin	GO:0010997;GO:0097027;GO:1990757
CDC23	Ubiquitin	GO:0004842
CDC34	Ubiquitin	GO:0004842;GO:0061631
CDC42	Ubiquitin	GO:0061630
CDKN1A	Ubiquitin	GO:0031625
CDKN1B	Ubiquitin	GO:0031625;GO:1990757
CEBPB	Ubiquitin	GO:0044389
CHEK2	Ubiquitin	GO:0031625
CHFR	Ubiquitin	GO:0004842;GO:0061630
CHMP3	Ubiquitin	GO:1990381
CKB	Ubiquitin	GO:0031625
CKS1B	Ubiquitin	GO:0043130
CKS2	Ubiquitin	GO:0043130
CLSPN	Ubiquitin	GO:0010997
CLTC	Ubiquitin	GO:1990381
CLU	Ubiquitin	GO:0031625
CNOT4	Ubiquitin	GO:0004842;GO:0061630
COP1	Ubiquitin	GO:0004842;GO:0061630
CRK	Ubiquitin	GO:0031625
CTNNB1	Ubiquitin	GO:0031625
CUEDC1	Ubiquitin	GO:0043130
CUEDC2	Ubiquitin	GO:0043130
CUL1	Ubiquitin	GO:0031625;GO:0160072
CUL2	Ubiquitin	GO:0004842;GO:0031625;GO:0160072
CUL3	Ubiquitin	GO:0004842;GO:0031625;GO:0061630;GO:0160072
CUL4A	Ubiquitin	GO:0031625;GO:0061630;GO:0160072
CUL4B	Ubiquitin	GO:0031625
CUL5	Ubiquitin	GO:0004842;GO:0031625;GO:0160072
CUL7	Ubiquitin	GO:0031625;GO:0160072
CUL9	Ubiquitin	GO:0004842;GO:0031625;GO:0061630
CXCR4	Ubiquitin	GO:0031625;GO:0043130
CYLD	Ubiquitin	GO:0004843;GO:0061578;GO:0061815;GO:1990380
DAW1	Ubiquitin	GO:1990756
DAXX	Ubiquitin	GO:0031625
DAZAP2	Ubiquitin	GO:0031625
DBT	Ubiquitin	GO:0031625
DCAF1	Ubiquitin	GO:1990756
DCAF12	Ubiquitin	GO:1990756
DCAF13	Ubiquitin	GO:1990756
DCST1	Ubiquitin	GO:0061630
DDB1	Ubiquitin	GO:0097602;GO:0160072
DDB2	Ubiquitin	GO:0004842
DDI2	Ubiquitin	GO:0043130
DERL1	Ubiquitin	GO:0031625;GO:1990381
DESI2	Ubiquitin	GO:0004843;GO:0061578;GO:0101005;GO:1990380
DET1	Ubiquitin	GO:0031625;GO:1990756
DHX16	Ubiquitin	GO:0043130
DIO2	Ubiquitin	GO:0031625
DLG3	Ubiquitin	GO:0031625
DNAAF10	Ubiquitin	GO:0043130
DNAJA1	Ubiquitin	GO:0031625;GO:0055131
DNAJB2	Ubiquitin	GO:0031593;GO:0031625;GO:0043130;GO:0140036
DNAJC2	Ubiquitin	GO:0061649
DNM1L	Ubiquitin	GO:0031625
DTL	Ubiquitin	GO:0004842
DTX1	Ubiquitin	GO:0031625;GO:0061630
DTX2	Ubiquitin	GO:0061630
DTX3	Ubiquitin	GO:0061630
DTX3L	Ubiquitin	GO:0004842;GO:0044389;GO:0061630;GO:0140852;GO:0141000
DTX4	Ubiquitin	GO:0004842;GO:0061630
DZIP3	Ubiquitin	GO:0004842;GO:0031593;GO:0061630
E4F1	Ubiquitin	GO:0061630
EGFR	Ubiquitin	GO:0031625
EIF3F	Ubiquitin	GO:0004843;GO:0101005;GO:0140492
EIF3H	Ubiquitin	GO:0140492
EIF4E2	Ubiquitin	GO:0031625
ELOB	Ubiquitin	GO:0031625
ENTREP1	Ubiquitin	GO:1990757
EPS15	Ubiquitin	GO:0031593;GO:0043130
ERBB3	Ubiquitin	GO:0031625
ERCC8	Ubiquitin	GO:1990756
ERLIN1	Ubiquitin	GO:0031625
ERLIN2	Ubiquitin	GO:0031625
FAAP20	Ubiquitin	GO:0031593;GO:0043130;GO:0070530;GO:0140036
FAF1	Ubiquitin	GO:0031625;GO:0043130
FAF2	Ubiquitin	GO:0031625;GO:0043130
FAN1	Ubiquitin	GO:0140036
FANCL	Ubiquitin	GO:0004842;GO:0031625;GO:0061630
FATE1	Ubiquitin	GO:0031625
FAU	Ubiquitin	GO:0031386;GO:0031625
FBXL14	Ubiquitin	GO:0004842
FBXL19	Ubiquitin	GO:1990756
FBXL2	Ubiquitin	GO:1990756
FBXL21P	Ubiquitin	GO:0004842
FBXL22	Ubiquitin	GO:0061630
FBXL3	Ubiquitin	GO:0004842
FBXL4	Ubiquitin	GO:1990756
FBXL5	Ubiquitin	GO:0004842
FBXL6	Ubiquitin	GO:0004842
FBXO10	Ubiquitin	GO:0004842
FBXO11	Ubiquitin	GO:0004842;GO:1990756
FBXO17	Ubiquitin	GO:0061630
FBXO2	Ubiquitin	GO:0004842;GO:0061630
FBXO21	Ubiquitin	GO:0004842
FBXO22	Ubiquitin	GO:0004842
FBXO24	Ubiquitin	GO:0004842
FBXO25	Ubiquitin	GO:0004842
FBXO27	Ubiquitin	GO:0061630
FBXO3	Ubiquitin	GO:0004842;GO:1990756
FBXO30	Ubiquitin	GO:0061630
FBXO31	Ubiquitin	GO:1990756
FBXO38	Ubiquitin	GO:1990756
FBXO4	Ubiquitin	GO:0004842;GO:0061630;GO:1990756
FBXO40	Ubiquitin	GO:0061630
FBXO42	Ubiquitin	GO:1990756
FBXO44	Ubiquitin	GO:0061630
FBXO45	Ubiquitin	GO:1990756
FBXO46	Ubiquitin	GO:1990756
FBXO5	Ubiquitin	GO:0010997;GO:1990948
FBXO6	Ubiquitin	GO:0061630
FBXO7	Ubiquitin	GO:0004842;GO:0031625;GO:0043130;GO:1990756
FBXO9	Ubiquitin	GO:0004842;GO:1990756
FBXW11	Ubiquitin	GO:1990756
FBXW2	Ubiquitin	GO:0004842
FBXW7	Ubiquitin	GO:0031625;GO:0043130;GO:0097027;GO:1990756
FBXW8	Ubiquitin	GO:1990756
FEM1A	Ubiquitin	GO:1990756
FEM1B	Ubiquitin	GO:1990756
FEM1C	Ubiquitin	GO:1990756
FHIT	Ubiquitin	GO:0031625
FOXL2	Ubiquitin	GO:0031624
FOXO1	Ubiquitin	GO:0031625
FZD4	Ubiquitin	GO:0031625
FZD5	Ubiquitin	GO:0031625
FZD6	Ubiquitin	GO:0031625
FZD8	Ubiquitin	GO:0031625
FZR1	Ubiquitin	GO:0010997;GO:0097027;GO:1990756;GO:1990757
G2E3	Ubiquitin	GO:0004842;GO:0061630
GABARAPL3	Ubiquitin	GO:0031625
GAN	Ubiquitin	GO:1990756
GCN1	Ubiquitin	GO:0055106
GGA1	Ubiquitin	GO:0043130
GGA2	Ubiquitin	GO:0043130
GGA3	Ubiquitin	GO:0043130
GGN	Ubiquitin	GO:0031625
GID4	Ubiquitin	GO:0061630
GLMN	Ubiquitin	GO:0031625;GO:0055105
GMCL2	Ubiquitin	GO:0097602
GPI	Ubiquitin	GO:0031625
GPR37	Ubiquitin	GO:0031625
GRIK2	Ubiquitin	GO:0031624;GO:0031625
GSK3B	Ubiquitin	GO:0031625
H2BC9	Ubiquitin	GO:0044389
HACE1	Ubiquitin	GO:0004842;GO:0061630
HAPSTR1	Ubiquitin	GO:0031625
HAPSTR2	Ubiquitin	GO:0031625
HDAC3	Ubiquitin	GO:1990381
HDAC6	Ubiquitin	GO:0031593;GO:0031625;GO:0043130
HECTD1	Ubiquitin	GO:0004842;GO:0061630
HECTD2	Ubiquitin	GO:0004842;GO:0061630
HECTD3	Ubiquitin	GO:0004842;GO:0061630
HECTD4	Ubiquitin	GO:0004842;GO:0061630
HECW1	Ubiquitin	GO:0004842;GO:0061630
HECW2	Ubiquitin	GO:0004842;GO:0061630
HERC1	Ubiquitin	GO:0004842;GO:0061630
HERC2P3	Ubiquitin	GO:0004842;GO:0061630
HERC3	Ubiquitin	GO:0004842;GO:0061630
HERC4	Ubiquitin	GO:0004842;GO:0061630
HERC6	Ubiquitin	GO:0004842;GO:0061630
HERPUD1	Ubiquitin	GO:1990756
HGS	Ubiquitin	GO:0043130;GO:0044389
HIF1A	Ubiquitin	GO:0031625
HLTF	Ubiquitin	GO:0031625;GO:0061630
HM13	Ubiquitin	GO:0031625
HSP90AA1	Ubiquitin	GO:0031625
HSP90AB1	Ubiquitin	GO:0031625
HSPA1A	Ubiquitin	GO:0031625;GO:0055131
HSPA1B	Ubiquitin	GO:0031625;GO:0055131
HSPA1L	Ubiquitin	GO:0031625
HSPA5	Ubiquitin	GO:0031625
HSPA8	Ubiquitin	GO:0031625;GO:0055131
HSPA9	Ubiquitin	GO:0031625
HSPB1	Ubiquitin	GO:0043130
HSPBP1	Ubiquitin	GO:0031625
HSPD1	Ubiquitin	GO:0031625
HTRA2	Ubiquitin	GO:1990948
HUWE1	Ubiquitin	GO:0004842;GO:0034450;GO:0061630;GO:0140852
IKBKE	Ubiquitin	GO:0031625;GO:0036435
IKBKG	Ubiquitin	GO:0031593;GO:0031625;GO:0070530;GO:1990450
ILRUN	Ubiquitin	GO:0043130
IPP	Ubiquitin	GO:1990756
IRF2BP1	Ubiquitin	GO:0061630
IRF2BPL	Ubiquitin	GO:0061630
ITCH	Ubiquitin	GO:0004842;GO:0019787;GO:0034450;GO:0044389;GO:0061630
IVNS1ABP	Ubiquitin	GO:1990756
JADE2	Ubiquitin	GO:0061630
JAK1	Ubiquitin	GO:0031625
JARID2	Ubiquitin	GO:0043130;GO:0061649
JKAMP	Ubiquitin	GO:0031625
JOSD1	Ubiquitin	GO:0004843;GO:0101005
JOSD2	Ubiquitin	GO:0004843;GO:0101005
JUN	Ubiquitin	GO:0031625;GO:0044389
KBTBD12	Ubiquitin	GO:1990756
KBTBD2	Ubiquitin	GO:1990756
KBTBD3	Ubiquitin	GO:1990756
KBTBD6	Ubiquitin	GO:1990756
KBTBD7	Ubiquitin	GO:1990756
KBTBD8	Ubiquitin	GO:1990756
KCMF1	Ubiquitin	GO:0061630
KCNH2	Ubiquitin	GO:0031625
KCNQ1	Ubiquitin	GO:0031625
KCTD10	Ubiquitin	GO:0004842
KCTD13	Ubiquitin	GO:0004842
KCTD17	Ubiquitin	GO:0097602;GO:1990756
KCTD2	Ubiquitin	GO:0097602
KCTD21	Ubiquitin	GO:0097602
KCTD5	Ubiquitin	GO:0097602
KCTD6	Ubiquitin	GO:0097602
KCTD9	Ubiquitin	GO:0097602
KDM4A	Ubiquitin	GO:0031625
KEAP1	Ubiquitin	GO:1990756
KLF1	Ubiquitin	GO:0043130
KLHDC1	Ubiquitin	GO:1990756
KLHDC10	Ubiquitin	GO:1990756
KLHDC2	Ubiquitin	GO:1990756
KLHDC3	Ubiquitin	GO:1990756
KLHL1	Ubiquitin	GO:1990756
KLHL10	Ubiquitin	GO:1990756
KLHL11	Ubiquitin	GO:1990756
KLHL12	Ubiquitin	GO:1990756
KLHL13	Ubiquitin	GO:0004842;GO:0097602
KLHL15	Ubiquitin	GO:1990756
KLHL17	Ubiquitin	GO:1990756
KLHL18	Ubiquitin	GO:1990756
KLHL2	Ubiquitin	GO:1990756
KLHL20	Ubiquitin	GO:0004842;GO:1990756
KLHL21	Ubiquitin	GO:0004842;GO:0097602;GO:1990756
KLHL22	Ubiquitin	GO:1990756
KLHL23	Ubiquitin	GO:1990756
KLHL24	Ubiquitin	GO:1990756
KLHL25	Ubiquitin	GO:1990756
KLHL28	Ubiquitin	GO:1990756
KLHL29	Ubiquitin	GO:1990756
KLHL3	Ubiquitin	GO:0097602;GO:1990756
KLHL30	Ubiquitin	GO:1990756
KLHL35	Ubiquitin	GO:1990756
KLHL36	Ubiquitin	GO:0097602
KLHL38	Ubiquitin	GO:1990756
KLHL4	Ubiquitin	GO:1990756
KLHL40	Ubiquitin	GO:1990756
KLHL41	Ubiquitin	GO:1990756
KLHL42	Ubiquitin	GO:0004842
KLHL5	Ubiquitin	GO:1990756
KLHL6	Ubiquitin	GO:1990756
KLHL7	Ubiquitin	GO:1990756
KLHL8	Ubiquitin	GO:1990756
KLHL9	Ubiquitin	GO:0004842;GO:0097602
LAPTM4B	Ubiquitin	GO:0031625
LAPTM5	Ubiquitin	GO:0031625
LCOR	Ubiquitin	GO:1990381
LIMK1	Ubiquitin	GO:0031625;GO:1990948
LMO7	Ubiquitin	GO:0004842
LNX1	Ubiquitin	GO:0004842;GO:0061630
LNX2	Ubiquitin	GO:0004842
LOC102724560	Ubiquitin	GO:0031625
LONRF2	Ubiquitin	GO:0061630
LRPPRC	Ubiquitin	GO:0031625
LRRC75A	Ubiquitin	GO:1990756
LRSAM1	Ubiquitin	GO:0004842;GO:0061630
LTBR	Ubiquitin	GO:0031625
LTN1	Ubiquitin	GO:0004842;GO:0061630
LYN	Ubiquitin	GO:0031625
MAEA	Ubiquitin	GO:0004842;GO:0061630
MAGEA2	Ubiquitin	GO:0031625
MAGEA2B	Ubiquitin	GO:0031625
MAGEC2	Ubiquitin	GO:0031625
MAGEL2	Ubiquitin	GO:0004842
MALT1	Ubiquitin	GO:0004842
MAP1LC3A	Ubiquitin	GO:0031625
MAP1LC3B	Ubiquitin	GO:0031625
MAP1LC3B2	Ubiquitin	GO:0031625
MAP1LC3C	Ubiquitin	GO:0031625
MAP3K7	Ubiquitin	GO:0031625;GO:1990450
MARCHF1	Ubiquitin	GO:0004842;GO:0061630
MARCHF10	Ubiquitin	GO:0061630
MARCHF11	Ubiquitin	GO:0004842;GO:0061630
MARCHF2	Ubiquitin	GO:0004842;GO:0061630
MARCHF3	Ubiquitin	GO:0004842;GO:0061630
MARCHF4	Ubiquitin	GO:0004842;GO:0061630
MARCHF5	Ubiquitin	GO:0061630
MARCHF6	Ubiquitin	GO:0004842;GO:0031624;GO:0061630;GO:1990381
MARCHF7	Ubiquitin	GO:0031624;GO:0043130;GO:0061630
MARCHF8	Ubiquitin	GO:0004842;GO:0061630
MARCHF9	Ubiquitin	GO:0004842;GO:0061630
MARK4	Ubiquitin	GO:0043130
MC1R	Ubiquitin	GO:0031625
MC4R	Ubiquitin	GO:0031625
MDM4	Ubiquitin	GO:0004842
MED1	Ubiquitin	GO:0061630
MED10	Ubiquitin	GO:0061630
MED11	Ubiquitin	GO:0061630
MED12	Ubiquitin	GO:0061630
MED17	Ubiquitin	GO:0061630
MED21	Ubiquitin	GO:0061630
MED27	Ubiquitin	GO:0061630
MED30	Ubiquitin	GO:0061630
MED31	Ubiquitin	GO:0061630
MED6	Ubiquitin	GO:0061630
MED7	Ubiquitin	GO:0061630
MED8	Ubiquitin	GO:0061630
MEFV	Ubiquitin	GO:0061630
MEX3C	Ubiquitin	GO:0061630
MFHAS1	Ubiquitin	GO:0031625
MFN2	Ubiquitin	GO:0031625
MGRN1	Ubiquitin	GO:0004842;GO:0061630
MIB1	Ubiquitin	GO:0004842;GO:0061630
MIB2	Ubiquitin	GO:0004842;GO:0061630
MID1	Ubiquitin	GO:0031625;GO:0061630
MID2	Ubiquitin	GO:0061630
MINDY1	Ubiquitin	GO:0004843;GO:0036435;GO:1990380
MINDY2	Ubiquitin	GO:0004843;GO:0036435;GO:0070530;GO:0071795;GO:0071796;GO:1990380
MINDY3	Ubiquitin	GO:0004843;GO:1990380
MINDY4	Ubiquitin	GO:0004843;GO:1990380
MINDY4B	Ubiquitin	GO:0004843;GO:1990380
MKRN1	Ubiquitin	GO:0061630
MKRN2	Ubiquitin	GO:0061630
MKRN3	Ubiquitin	GO:0061630
MKRN4P	Ubiquitin	GO:0061630
MOAP1	Ubiquitin	GO:0031625
MPND	Ubiquitin	GO:0031593;GO:0140492
MSL2	Ubiquitin	GO:0061630;GO:0141054
MVB12A	Ubiquitin	GO:0043130
MYC	Ubiquitin	GO:0031625;GO:1905761
MYCBP2	Ubiquitin	GO:0061630
MYLIP	Ubiquitin	GO:0004842;GO:0061630
MYOD1	Ubiquitin	GO:0031625
MYSM1	Ubiquitin	GO:0140492;GO:0140950
N4BP1	Ubiquitin	GO:0043130
N4BP2	Ubiquitin	GO:0043130
NBR1	Ubiquitin	GO:0043130
NCCRP1	Ubiquitin	GO:0061630
NDUFS2	Ubiquitin	GO:0031625
NEDD4	Ubiquitin	GO:0004842;GO:0043130;GO:0061630
NEDD4L	Ubiquitin	GO:0004842;GO:0061630
NEK6	Ubiquitin	GO:0031625
NEURL1	Ubiquitin	GO:0004842;GO:0061630
NEURL1B	Ubiquitin	GO:0061630
NEURL2	Ubiquitin	GO:0061630
NEURL3	Ubiquitin	GO:0061630
NEURL4	Ubiquitin	GO:0061630
NFE2L2	Ubiquitin	GO:0031625
NFKBIA	Ubiquitin	GO:0031625
NFX1	Ubiquitin	GO:0061630
NGFR	Ubiquitin	GO:0031625
NHERF4	Ubiquitin	GO:1990381
NHLRC1	Ubiquitin	GO:0004842;GO:0061630
NHLRC3	Ubiquitin	GO:0061630
NKD2	Ubiquitin	GO:0031625
NLK	Ubiquitin	GO:0031625
NOD1	Ubiquitin	GO:0043130
NOD2	Ubiquitin	GO:0043130
NOSIP	Ubiquitin	GO:0061630
NPLOC4	Ubiquitin	GO:0031625;GO:0036435;GO:0043130;GO:0070530
NSFL1C	Ubiquitin	GO:0043130
NT5C2	Ubiquitin	GO:0061630
NUP62	Ubiquitin	GO:0043130
OBI1	Ubiquitin	GO:0004842;GO:0061630
OPTN	Ubiquitin	GO:0031593;GO:0070530
OTUB2	Ubiquitin	GO:0004843;GO:0043130
OTUD1	Ubiquitin	GO:0004843
OTUD3	Ubiquitin	GO:0004843
OTUD4	Ubiquitin	GO:0004843;GO:0061578
OTUD5	Ubiquitin	GO:0004843;GO:0061578;GO:0101005;GO:1990380
OTUD6A	Ubiquitin	GO:0004843
OTUD6B	Ubiquitin	GO:0004843
OTUD7A	Ubiquitin	GO:0004843;GO:0070530
OTUD7B	Ubiquitin	GO:0004843;GO:0070530;GO:1990380
OTULIN	Ubiquitin	GO:0004843
PA2G4	Ubiquitin	GO:0031625
PACRG	Ubiquitin	GO:0031625
PAQR3	Ubiquitin	GO:1990756
PARK7	Ubiquitin	GO:0044388;GO:0044390;GO:0055105;GO:1990381
PARP1	Ubiquitin	GO:0031625
PARP10	Ubiquitin	GO:0070530
PARP9	Ubiquitin	GO:0044389
PATZ1	Ubiquitin	GO:0031625
PAX6	Ubiquitin	GO:0031625
PCBP2	Ubiquitin	GO:0031625
PCGF3	Ubiquitin	GO:0140862
PCGF5	Ubiquitin	GO:0140862
PCMTD1	Ubiquitin	GO:1990756
PDCD6	Ubiquitin	GO:1990756
PDLIM2	Ubiquitin	GO:0031625
PDZRN3	Ubiquitin	GO:0004842;GO:0061630
PEF1	Ubiquitin	GO:1990756
PELI1	Ubiquitin	GO:0004842;GO:0034450;GO:0061630
PELI2	Ubiquitin	GO:0034450;GO:0061630
PELI3	Ubiquitin	GO:0061630
PER1	Ubiquitin	GO:0031625
PER3	Ubiquitin	GO:0031625
PEX1	Ubiquitin	GO:0140036
PEX10	Ubiquitin	GO:0061630
PEX12	Ubiquitin	GO:0004842;GO:1990757
PEX2	Ubiquitin	GO:0061630
PEX6	Ubiquitin	GO:0140036
PIN1	Ubiquitin	GO:1990757
PINK1	Ubiquitin	GO:0031625;GO:0055131
PJA1	Ubiquitin	GO:0061630
PJA2	Ubiquitin	GO:0004842;GO:0061630
PLAA	Ubiquitin	GO:0043130
PLK1	Ubiquitin	GO:0010997
POLR2A	Ubiquitin	GO:0031625
POU5F1	Ubiquitin	GO:0031625
PPARA	Ubiquitin	GO:0031624
PPARGC1A	Ubiquitin	GO:0031625
PPIL2	Ubiquitin	GO:0004842;GO:0034450;GO:0061630
PPP1R11	Ubiquitin	GO:0061630
PRAME	Ubiquitin	GO:1990756
PRAMEF1	Ubiquitin	GO:1990756
PRAMEF10	Ubiquitin	GO:1990756
PRAMEF11	Ubiquitin	GO:1990756
PRAMEF12	Ubiquitin	GO:1990756
PRAMEF13	Ubiquitin	GO:1990756
PRAMEF14	Ubiquitin	GO:1990756
PRAMEF15	Ubiquitin	GO:1990756
PRAMEF17	Ubiquitin	GO:1990756
PRAMEF18	Ubiquitin	GO:1990756
PRAMEF19	Ubiquitin	GO:1990756
PRAMEF2	Ubiquitin	GO:1990756
PRAMEF20	Ubiquitin	GO:1990756
PRAMEF22	Ubiquitin	GO:1990756
PRAMEF25	Ubiquitin	GO:1990756
PRAMEF26	Ubiquitin	GO:1990756
PRAMEF27	Ubiquitin	GO:1990756
PRAMEF33	Ubiquitin	GO:1990756
PRAMEF4	Ubiquitin	GO:1990756
PRAMEF5	Ubiquitin	GO:1990756
PRAMEF6	Ubiquitin	GO:1990756
PRAMEF7	Ubiquitin	GO:1990756
PRAMEF8	Ubiquitin	GO:1990756
PRAMEF9	Ubiquitin	GO:1990756
PRDX6	Ubiquitin	GO:0031625
PRKACA	Ubiquitin	GO:0031625
PRKACB	Ubiquitin	GO:0031625
PRKAR1A	Ubiquitin	GO:0031625
PRKAR2A	Ubiquitin	GO:0031625
PRKAR2B	Ubiquitin	GO:0031625
PRKN	Ubiquitin	GO:0004842;GO:0031624;GO:0031625;GO:0043130;GO:0061630;GO:0097602;GO:1990381
PRPF19	Ubiquitin	GO:0004842;GO:0034450;GO:0061630
PRPF8	Ubiquitin	GO:0070530
PRR5L	Ubiquitin	GO:0031625
PRR7	Ubiquitin	GO:0044389
PSMA3	Ubiquitin	GO:0031625
PSMD1	Ubiquitin	GO:0031625
PSMD14	Ubiquitin	GO:0061578;GO:0140492
PSMD4	Ubiquitin	GO:0031593
PTEN	Ubiquitin	GO:0010997;GO:1990381;GO:1990757
PTPN22	Ubiquitin	GO:0031625
PTPRN	Ubiquitin	GO:0044389
PYHIN1	Ubiquitin	GO:0031625
RAB40A	Ubiquitin	GO:1990756
RAB40B	Ubiquitin	GO:1990756
RAB40C	Ubiquitin	GO:1990756
RABGEF1	Ubiquitin	GO:0061630
RAD18	Ubiquitin	GO:0031593;GO:0031625;GO:0061630
RAD23A	Ubiquitin	GO:0031593;GO:0043130;GO:1990381
RAD23B	Ubiquitin	GO:0031593;GO:0043130
RAE1	Ubiquitin	GO:0043130
RAG1	Ubiquitin	GO:0004842;GO:0061630
RALA	Ubiquitin	GO:0031625
RALB	Ubiquitin	GO:0031625
RB1	Ubiquitin	GO:0031625
RBBP6	Ubiquitin	GO:0004842;GO:0061630
RBCK1	Ubiquitin	GO:0004842;GO:0043130;GO:0061630;GO:1990757
RC3H1	Ubiquitin	GO:0004842;GO:0061630
RC3H2	Ubiquitin	GO:0061630
RCHY1	Ubiquitin	GO:0004842;GO:0061630
RELA	Ubiquitin	GO:0031625
RFFL	Ubiquitin	GO:0031625;GO:0061630
RFPL1	Ubiquitin	GO:0061630
RFPL2	Ubiquitin	GO:0061630
RFPL3	Ubiquitin	GO:0061630
RFPL4A	Ubiquitin	GO:0061630
RFPL4AL1	Ubiquitin	GO:0061630
RFPL4B	Ubiquitin	GO:0061630
RFWD3	Ubiquitin	GO:0004842;GO:0061630
RHOBTB2	Ubiquitin	GO:1990756
RHOBTB3	Ubiquitin	GO:0031625
RIGI	Ubiquitin	GO:0031625
RING1	Ubiquitin	GO:0061630;GO:0097027
RIPK1	Ubiquitin	GO:0031625
RLIM	Ubiquitin	GO:0004842;GO:0061630
RMND5A	Ubiquitin	GO:0004842;GO:0061630
RMND5B	Ubiquitin	GO:0004842;GO:0061630
RNF10	Ubiquitin	GO:0061630
RNF103	Ubiquitin	GO:0004842;GO:0061630
RNF11	Ubiquitin	GO:0061630
RNF112	Ubiquitin	GO:0061630
RNF113A	Ubiquitin	GO:0061630
RNF114	Ubiquitin	GO:0061630
RNF115	Ubiquitin	GO:0004842;GO:0061630
RNF121	Ubiquitin	GO:0061630
RNF122	Ubiquitin	GO:0061630
RNF123	Ubiquitin	GO:0004842;GO:0061630
RNF125	Ubiquitin	GO:0004842;GO:0031624;GO:0061630
RNF126	Ubiquitin	GO:0061630
RNF128	Ubiquitin	GO:0061630
RNF13	Ubiquitin	GO:0004842;GO:0061630
RNF130	Ubiquitin	GO:0004842;GO:0061630
RNF133	Ubiquitin	GO:0061630
RNF135	Ubiquitin	GO:0004842;GO:0061630
RNF138	Ubiquitin	GO:0061630
RNF139	Ubiquitin	GO:0004842;GO:0019787;GO:0061630
RNF14	Ubiquitin	GO:0004842;GO:0019787;GO:0031624;GO:0061630
RNF141	Ubiquitin	GO:0004842
RNF144A	Ubiquitin	GO:0004842;GO:0031624;GO:0061630
RNF144B	Ubiquitin	GO:0004842;GO:0031624;GO:0061630
RNF145	Ubiquitin	GO:0061630
RNF146	Ubiquitin	GO:0004842;GO:0061630
RNF148	Ubiquitin	GO:0061630
RNF149	Ubiquitin	GO:0061630
RNF150	Ubiquitin	GO:0061630
RNF152	Ubiquitin	GO:0004842;GO:0061630
RNF157	Ubiquitin	GO:0004842;GO:0061630
RNF166	Ubiquitin	GO:0061630
RNF167	Ubiquitin	GO:0004842;GO:0061630
RNF168	Ubiquitin	GO:0004842;GO:0043130;GO:0061630;GO:0070530;GO:0140852;GO:0140858
RNF169	Ubiquitin	GO:0004842;GO:0061630;GO:0061649;GO:0070530
RNF170	Ubiquitin	GO:0061630
RNF175	Ubiquitin	GO:0061630
RNF180	Ubiquitin	GO:0031624;GO:0061630
RNF181	Ubiquitin	GO:0004842;GO:0061630
RNF182	Ubiquitin	GO:0004842;GO:0061630
RNF183	Ubiquitin	GO:0061630
RNF185	Ubiquitin	GO:0004842;GO:0043130;GO:0044390;GO:0061630
RNF186	Ubiquitin	GO:0004842;GO:0031625;GO:0061630
RNF187	Ubiquitin	GO:0004842;GO:0061630
RNF19A	Ubiquitin	GO:0004842;GO:0031624;GO:0061630
RNF19B	Ubiquitin	GO:0004842;GO:0031624;GO:0043130;GO:0061630
RNF2	Ubiquitin	GO:0004842;GO:0061630;GO:0140862
RNF20	Ubiquitin	GO:0004842;GO:0031625;GO:0061630;GO:0140850
RNF208	Ubiquitin	GO:0004842;GO:0061630
RNF213	Ubiquitin	GO:0004842;GO:0061630
RNF214	Ubiquitin	GO:0004842
RNF215	Ubiquitin	GO:0061630
RNF216	Ubiquitin	GO:0061630
RNF217	Ubiquitin	GO:0004842;GO:0031624;GO:0061630
RNF220	Ubiquitin	GO:0004842;GO:0061630
RNF223	Ubiquitin	GO:0061630
RNF225	Ubiquitin	GO:0061630
RNF227	Ubiquitin	GO:0061630
RNF228	Ubiquitin	GO:0061630
RNF25	Ubiquitin	GO:0004842;GO:0061630
RNF26	Ubiquitin	GO:0061630
RNF31	Ubiquitin	GO:0004842;GO:0031625;GO:0036435;GO:0043130;GO:0061630;GO:0070530;GO:1990450
RNF34	Ubiquitin	GO:0031625;GO:0061630
RNF38	Ubiquitin	GO:0061630
RNF39	Ubiquitin	GO:0061630
RNF40	Ubiquitin	GO:0004842;GO:0031625;GO:0061630
RNF41	Ubiquitin	GO:0004842;GO:0061630
RNF43	Ubiquitin	GO:0004842;GO:0061630
RNF44	Ubiquitin	GO:0061630
RNF5	Ubiquitin	GO:0004842;GO:0044390;GO:0061630
RNF6	Ubiquitin	GO:0004842;GO:0061630
RNF8	Ubiquitin	GO:0004842;GO:0031625;GO:0043130;GO:0061630
RNFT1	Ubiquitin	GO:0043130;GO:0061630
RNFT2	Ubiquitin	GO:0061630
RPA2	Ubiquitin	GO:0031625
RPGR	Ubiquitin	GO:0061630
RPL11	Ubiquitin	GO:0031625;GO:1990948
RPL23	Ubiquitin	GO:0031625;GO:1990948
RPL37	Ubiquitin	GO:1990948
RPL5	Ubiquitin	GO:0031625;GO:1990948
RPS15	Ubiquitin	GO:1990948
RPS20	Ubiquitin	GO:1990948
RPS27A	Ubiquitin	GO:0031386;GO:0031625
RPS3	Ubiquitin	GO:0044390
RPS7	Ubiquitin	GO:1990948
RRAGA	Ubiquitin	GO:0031625
RSPRY1	Ubiquitin	GO:0004842
RTN4	Ubiquitin	GO:0031625
SART3	Ubiquitin	GO:1990381
SCAMP3	Ubiquitin	GO:0031625
SCN5A	Ubiquitin	GO:0031625
SELENOS	Ubiquitin	GO:1990381
SH3BGRL	Ubiquitin	GO:1990756
SH3KBP1	Ubiquitin	GO:0031625
SH3RF1	Ubiquitin	GO:0061630
SH3RF2	Ubiquitin	GO:0061630
SH3RF3	Ubiquitin	GO:0061630
SHARPIN	Ubiquitin	GO:0004842;GO:0031593;GO:0043130
SHPRH	Ubiquitin	GO:0004842;GO:0031625;GO:0061630
SIAH1	Ubiquitin	GO:0004842;GO:0031624;GO:0061630
SIAH2	Ubiquitin	GO:0004842;GO:0031624;GO:0061630
SIAH3	Ubiquitin	GO:0031624;GO:0061630
SIRT2	Ubiquitin	GO:0043130
SKI	Ubiquitin	GO:0031625
SKP1	Ubiquitin	GO:0097602;GO:0160072;GO:1990756;GO:1990757
SKP2	Ubiquitin	GO:1990756
SLC25A5	Ubiquitin	GO:0031625
SLC67A1	Ubiquitin	GO:0031625
SMAD1	Ubiquitin	GO:0031625
SMAD2	Ubiquitin	GO:0031625
SMAD3	Ubiquitin	GO:0031625;GO:0043130
SMAD5	Ubiquitin	GO:0031625
SMAD6	Ubiquitin	GO:0031625
SMAD7	Ubiquitin	GO:0031625;GO:1990756
SMARCAD1	Ubiquitin	GO:0043130
SMG5	Ubiquitin	GO:0031625
SMURF1	Ubiquitin	GO:0004842;GO:0061630
SMURF2	Ubiquitin	GO:0004842;GO:0061630
SNCAIP	Ubiquitin	GO:0031625
SNX9	Ubiquitin	GO:0031625
SOCS2	Ubiquitin	GO:1990756
SOCS7	Ubiquitin	GO:1990756
SPART	Ubiquitin	GO:0031625
SPATA2	Ubiquitin	GO:1990381
SPOP	Ubiquitin	GO:0031625
SPOPL	Ubiquitin	GO:0031625
SPRTN	Ubiquitin	GO:0031593;GO:0043130;GO:0070530
SPRY2	Ubiquitin	GO:0055105
SPSB1	Ubiquitin	GO:1990756
SPSB2	Ubiquitin	GO:1990756
SPSB3	Ubiquitin	GO:1990756
SPSB4	Ubiquitin	GO:1990756
SQSTM1	Ubiquitin	GO:0031625;GO:0043130;GO:0070530;GO:0140036
STAM	Ubiquitin	GO:0043130;GO:0044389
STAM2	Ubiquitin	GO:0043130
STAMBP	Ubiquitin	GO:0061578;GO:0101005;GO:0140492
STAMBPL1	Ubiquitin	GO:0061578;GO:0140492
STAT1	Ubiquitin	GO:0044389
STAT2	Ubiquitin	GO:0044389
STING1	Ubiquitin	GO:0031625
STUB1	Ubiquitin	GO:0004842;GO:0031625;GO:0034450;GO:0061630
STX8	Ubiquitin	GO:0031625
SUV39H2	Ubiquitin	GO:1990756
SYT11	Ubiquitin	GO:0031625
SYVN1	Ubiquitin	GO:0061630;GO:1990381
Table 2	Ubiquitin	GO:0043130;GO:0070530
Table 3	Ubiquitin	GO:0043130;GO:0070530
TAF1	Ubiquitin	GO:0061631
TANK	Ubiquitin	GO:0004843;GO:0031625
TCERG1	Ubiquitin	GO:0044390
TCP1	Ubiquitin	GO:0031625
TGFBR1	Ubiquitin	GO:0031625
TLR3	Ubiquitin	GO:0044389
TMBIM6	Ubiquitin	GO:0031625
TMEM129	Ubiquitin	GO:0061630
TNFAIP1	Ubiquitin	GO:0004842
TNFAIP3	Ubiquitin	GO:0004842;GO:0004843;GO:0043130;GO:0061578;GO:0070530
TNFRSF14	Ubiquitin	GO:0031625
TNFRSF1B	Ubiquitin	GO:0031625
TNIP2	Ubiquitin	GO:0031593;GO:0070530
TNIP3	Ubiquitin	GO:0031593
TNK2	Ubiquitin	GO:0031625
TOM1	Ubiquitin	GO:0031593;GO:0043130
TOM1L1	Ubiquitin	GO:0043130
TOM1L2	Ubiquitin	GO:0043130
TOP2A	Ubiquitin	GO:0043130
TP53	Ubiquitin	GO:0031625
TP53BP1	Ubiquitin	GO:0061649
TP53INP2	Ubiquitin	GO:0043130
TPI1	Ubiquitin	GO:0031625
TRAF1	Ubiquitin	GO:0031625
TRAF2	Ubiquitin	GO:0004842;GO:0031625;GO:0061630
TRAF3	Ubiquitin	GO:0004842;GO:0031625;GO:0061630
TRAF3IP2	Ubiquitin	GO:0061630
TRAF4	Ubiquitin	GO:0031625
TRAF5	Ubiquitin	GO:0031625;GO:0061630
TRAF6	Ubiquitin	GO:0004842;GO:0031624;GO:0034450;GO:0061630
TRAF7	Ubiquitin	GO:0004842;GO:0061630
TRAIP	Ubiquitin	GO:0004842;GO:0061630
TRIB1	Ubiquitin	GO:0031625;GO:0055106
TRIB2	Ubiquitin	GO:0031625;GO:0055106
TRIB3	Ubiquitin	GO:0031625;GO:0055106;GO:1990757
TRIM10	Ubiquitin	GO:0061630
TRIM11	Ubiquitin	GO:0004842;GO:0061630
TRIM13	Ubiquitin	GO:0004842;GO:0061630;GO:0061659
TRIM14	Ubiquitin	GO:0061630
TRIM15	Ubiquitin	GO:0061630
TRIM16	Ubiquitin	GO:0061630
TRIM17	Ubiquitin	GO:0004842;GO:0061630
TRIM2	Ubiquitin	GO:0004842;GO:0061630
TRIM21	Ubiquitin	GO:0004842;GO:0061630
TRIM22	Ubiquitin	GO:0061630
TRIM23	Ubiquitin	GO:0004842;GO:0061630
TRIM24	Ubiquitin	GO:0004842;GO:0061630
TRIM25	Ubiquitin	GO:0004842;GO:0061630
TRIM26	Ubiquitin	GO:0061630
TRIM3	Ubiquitin	GO:0004842;GO:0061630
TRIM31	Ubiquitin	GO:0061630
TRIM32	Ubiquitin	GO:0004842;GO:0043130;GO:0061630
TRIM33	Ubiquitin	GO:0004842;GO:0061630
TRIM34	Ubiquitin	GO:0061630
TRIM35	Ubiquitin	GO:0061630
TRIM36	Ubiquitin	GO:0004842;GO:0061630
TRIM37	Ubiquitin	GO:0004842;GO:0031625;GO:0061630;GO:0140862
TRIM39	Ubiquitin	GO:0061630
TRIM4	Ubiquitin	GO:0004842;GO:0061630
TRIM41	Ubiquitin	GO:0061630
TRIM42	Ubiquitin	GO:0061630
TRIM43	Ubiquitin	GO:0061630
TRIM43B	Ubiquitin	GO:0061630
TRIM44	Ubiquitin	GO:0061630
TRIM45	Ubiquitin	GO:0061630
TRIM47	Ubiquitin	GO:0004842;GO:0061630
TRIM48	Ubiquitin	GO:0061630
TRIM49	Ubiquitin	GO:0061630
TRIM49B	Ubiquitin	GO:0061630
TRIM49C	Ubiquitin	GO:0061630
TRIM49D1	Ubiquitin	GO:0061630
TRIM49D2	Ubiquitin	GO:0061630
TRIM5	Ubiquitin	GO:0004842;GO:0061630
TRIM50	Ubiquitin	GO:0061630
TRIM51	Ubiquitin	GO:0061630
TRIM51G	Ubiquitin	GO:0061630
TRIM52	Ubiquitin	GO:0061630
TRIM54	Ubiquitin	GO:0061630
TRIM55	Ubiquitin	GO:0061630
TRIM56	Ubiquitin	GO:0004842;GO:0061630
TRIM58	Ubiquitin	GO:0061630
TRIM59	Ubiquitin	GO:0061630
TRIM6	Ubiquitin	GO:0061630
TRIM61	Ubiquitin	GO:0061630
TRIM62	Ubiquitin	GO:0004842;GO:0061630
TRIM63	Ubiquitin	GO:0004842;GO:0061630
TRIM64	Ubiquitin	GO:0061630
TRIM64B	Ubiquitin	GO:0061630
TRIM64C	Ubiquitin	GO:0061630
TRIM65	Ubiquitin	GO:0004842;GO:0061630
TRIM68	Ubiquitin	GO:0004842;GO:0061630
TRIM69	Ubiquitin	GO:0004842;GO:0061630
TRIM7	Ubiquitin	GO:0061630
TRIM71	Ubiquitin	GO:0004842;GO:0061630
TRIM72	Ubiquitin	GO:0031624;GO:0061630
TRIM73	Ubiquitin	GO:0061630
TRIM74	Ubiquitin	GO:0061630
TRIM75	Ubiquitin	GO:0061630
TRIM77	Ubiquitin	GO:0061630
TRIM8	Ubiquitin	GO:0061630
TRIM9	Ubiquitin	GO:0061630
TRIML1	Ubiquitin	GO:0061630
TRIML2	Ubiquitin	GO:0061630
TRIOBP	Ubiquitin	GO:0031625
TRIP12	Ubiquitin	GO:0004842;GO:0061630
TRIP4	Ubiquitin	GO:0044389
TRPC4AP	Ubiquitin	GO:1990756
TSG101	Ubiquitin	GO:0031625;GO:0043130
TTC3	Ubiquitin	GO:0004842;GO:0061630
TUBA1B	Ubiquitin	GO:0031625
TUBB	Ubiquitin	GO:0031625
TXNIP	Ubiquitin	GO:0031625
UBA1	Ubiquitin	GO:0004839;GO:0008641
UBA52	Ubiquitin	GO:0031386;GO:0031625
UBAC1	Ubiquitin	GO:0031593
UBAP1	Ubiquitin	GO:0043130
UBAP1L	Ubiquitin	GO:0043130
UBASH3B	Ubiquitin	GO:0031625
UBB	Ubiquitin	GO:0031386;GO:0031625
UBC	Ubiquitin	GO:0031386;GO:0031625
UBE2A	Ubiquitin	GO:0004842;GO:0019787;GO:0031625;GO:0043130;GO:0061631
UBE2B	Ubiquitin	GO:0004842;GO:0019787;GO:0031625;GO:0061631
UBE2C	Ubiquitin	GO:0004842;GO:0019787;GO:0044389;GO:0061630;GO:0061631
UBE2D1	Ubiquitin	GO:0004842;GO:0061630;GO:0061631
UBE2D2	Ubiquitin	GO:0004842;GO:0061630;GO:0061631
UBE2D3	Ubiquitin	GO:0004842;GO:0061630;GO:0061631
UBE2D4	Ubiquitin	GO:0004842;GO:0061631
UBE2E3	Ubiquitin	GO:0004842;GO:0061631
UBE2G1	Ubiquitin	GO:0004842;GO:0019787;GO:0031625;GO:0061631
UBE2G2	Ubiquitin	GO:0004842;GO:0061631
UBE2H	Ubiquitin	GO:0004842;GO:0061631
UBE2J1	Ubiquitin	GO:0031625;GO:0061631
UBE2J2	Ubiquitin	GO:0031625;GO:0061631
UBE2K	Ubiquitin	GO:0004842;GO:0031625;GO:0034450;GO:0061631
UBE2L3	Ubiquitin	GO:0004842;GO:0019787;GO:0031625;GO:0061631;GO:0097027
UBE2L5	Ubiquitin	GO:0019787;GO:0061631
UBE2N	Ubiquitin	GO:0004842;GO:0031625;GO:0043130;GO:0061631;GO:0097027
UBE2NL	Ubiquitin	GO:0061631
UBE2O	Ubiquitin	GO:0004842;GO:0061630;GO:0061631
UBE2Q1	Ubiquitin	GO:0004842;GO:0061631
UBE2Q2	Ubiquitin	GO:0004842;GO:0061631
UBE2QL1	Ubiquitin	GO:0061631
UBE2R2	Ubiquitin	GO:0004842;GO:0061631
UBE2S	Ubiquitin	GO:0004842;GO:0010997;GO:0019787;GO:0061631
UBE2T	Ubiquitin	GO:0004842;GO:0019787;GO:0031625;GO:0061631
UBE2U	Ubiquitin	GO:0019787;GO:0061631
UBE2V1	Ubiquitin	GO:0031624;GO:0061631
UBE2W	Ubiquitin	GO:0004842;GO:0031625;GO:0061631
UBE3A	Ubiquitin	GO:0004842;GO:0061630
UBE3B	Ubiquitin	GO:0004842;GO:0061630
UBE3C	Ubiquitin	GO:0004842;GO:0061630
UBE3D	Ubiquitin	GO:0031624;GO:0044390;GO:0061630
UBE4A	Ubiquitin	GO:0004842;GO:0034450;GO:0061630
UBE4B	Ubiquitin	GO:0004842;GO:0034450;GO:0061630
UBL4A	Ubiquitin	GO:0019787
UBL5	Ubiquitin	GO:0031386
UBL7	Ubiquitin	GO:0031593
UBOX5	Ubiquitin	GO:0004842;GO:0031625;GO:0034450;GO:0061630
UBQLN1	Ubiquitin	GO:0031593
UBQLN2	Ubiquitin	GO:0031593
UBQLN3	Ubiquitin	GO:0031593
UBQLN4	Ubiquitin	GO:0031593;GO:0036435
UBQLNL	Ubiquitin	GO:0031593
UBR1	Ubiquitin	GO:0004842;GO:0061630
UBR2	Ubiquitin	GO:0061630;GO:0141053
UBR3	Ubiquitin	GO:0004842;GO:0061630
UBR4	Ubiquitin	GO:0004842;GO:0061630;GO:1990756
UBR5	Ubiquitin	GO:0004842;GO:0034450;GO:0043130;GO:0061630
UBR7	Ubiquitin	GO:0061630
UBXN1	Ubiquitin	GO:0031593;GO:0031625;GO:0036435;GO:0043130;GO:0071796
UBXN10	Ubiquitin	GO:0043130
UBXN11	Ubiquitin	GO:0043130
UBXN2A	Ubiquitin	GO:0043130
UBXN2B	Ubiquitin	GO:0043130
UBXN7	Ubiquitin	GO:0031625;GO:0043130
UBXN8	Ubiquitin	GO:0043130
UCHL1	Ubiquitin	GO:0004843;GO:0031625;GO:0043130
UCHL5	Ubiquitin	GO:0004843
UEVLD	Ubiquitin	GO:0043130
UFD1	Ubiquitin	GO:0004843;GO:0031593;GO:0036435
UHRF1	Ubiquitin	GO:0004842;GO:0061630;GO:0141055
UIMC1	Ubiquitin	GO:0061649;GO:0070530
UQCRC1	Ubiquitin	GO:0031625
USP1	Ubiquitin	GO:0004843
USP10	Ubiquitin	GO:0004843
USP11	Ubiquitin	GO:0004843
USP12	Ubiquitin	GO:0004843;GO:0101005
USP13	Ubiquitin	GO:0004843;GO:0031625;GO:0043130;GO:0044389;GO:1990380
USP14	Ubiquitin	GO:0004843;GO:0061578;GO:0101005
USP15	Ubiquitin	GO:0004843;GO:0061649;GO:0101005;GO:1990380
USP16	Ubiquitin	GO:0004843;GO:0043130;GO:0140950
USP17L1	Ubiquitin	GO:0004843
USP17L10	Ubiquitin	GO:0004843
USP17L11	Ubiquitin	GO:0004843
USP17L12	Ubiquitin	GO:0004843
USP17L13	Ubiquitin	GO:0004843
USP17L15	Ubiquitin	GO:0004843
USP17L17	Ubiquitin	GO:0004843
USP17L18	Ubiquitin	GO:0004843
USP17L19	Ubiquitin	GO:0004843
USP17L2	Ubiquitin	GO:0004843
USP17L20	Ubiquitin	GO:0004843
USP17L21	Ubiquitin	GO:0004843
USP17L22	Ubiquitin	GO:0004843
USP17L23	Ubiquitin	GO:0004843
USP17L24	Ubiquitin	GO:0004843
USP17L3	Ubiquitin	GO:0004843
USP17L4	Ubiquitin	GO:0004843
USP17L5	Ubiquitin	GO:0004843
USP17L6P	Ubiquitin	GO:0004843
USP17L7	Ubiquitin	GO:0004843
USP17L8	Ubiquitin	GO:0004843
USP19	Ubiquitin	GO:0004843
USP2	Ubiquitin	GO:0004843;GO:0031625
USP20	Ubiquitin	GO:0004843
USP22	Ubiquitin	GO:0004843;GO:0140936;GO:0140950
USP24	Ubiquitin	GO:0004843
USP26	Ubiquitin	GO:0004843;GO:0101005;GO:1990380
USP27X	Ubiquitin	GO:0004843;GO:0061578;GO:1990380
USP28	Ubiquitin	GO:0004843;GO:0101005
USP29	Ubiquitin	GO:0004843;GO:0101005
USP3	Ubiquitin	GO:0004843;GO:0140936;GO:0140950
USP30	Ubiquitin	GO:0004843;GO:0101005
USP31	Ubiquitin	GO:0004843;GO:0019783
USP32	Ubiquitin	GO:0004843
USP33	Ubiquitin	GO:0004843
USP34	Ubiquitin	GO:0004843
USP35	Ubiquitin	GO:0004843
USP36	Ubiquitin	GO:0004843;GO:0140936;GO:1990380
USP37	Ubiquitin	GO:0004843;GO:0101005
USP38	Ubiquitin	GO:0004843
USP4	Ubiquitin	GO:0004843
USP40	Ubiquitin	GO:0004843
USP42	Ubiquitin	GO:0004843
USP44	Ubiquitin	GO:0004843;GO:0101005
USP45	Ubiquitin	GO:0004843
USP46	Ubiquitin	GO:0004843;GO:0101005
USP47	Ubiquitin	GO:0004843;GO:0101005
USP48	Ubiquitin	GO:0004843;GO:0101005
USP49	Ubiquitin	GO:0004843;GO:0140936
USP5	Ubiquitin	GO:0004843;GO:0043130;GO:0101005
USP50	Ubiquitin	GO:0004843;GO:0019783
USP51	Ubiquitin	GO:0004843;GO:0140950
USP53	Ubiquitin	GO:0061578
USP54	Ubiquitin	GO:0004843;GO:0061578
USP6	Ubiquitin	GO:0004843
USP7	Ubiquitin	GO:0004843;GO:0101005;GO:1990380
USP8	Ubiquitin	GO:0004843;GO:0061578;GO:1990380
USP9X	Ubiquitin	GO:0004843;GO:0061578;GO:0101005;GO:0180017;GO:1990380
USP9Y	Ubiquitin	GO:0004843
VCL	Ubiquitin	GO:0031625
VCP	Ubiquitin	GO:0031593;GO:0031625;GO:0036435;GO:0044389;GO:0140036;GO:1990381
VCPIP1	Ubiquitin	GO:0004843
VHL	Ubiquitin	GO:0004842;GO:1990756
VPS11	Ubiquitin	GO:0061630
VPS18	Ubiquitin	GO:0061630
VPS28	Ubiquitin	GO:0043130
VPS36	Ubiquitin	GO:0043130
WASHC1	Ubiquitin	GO:0031625
WBP1L	Ubiquitin	GO:0031625
WDR24	Ubiquitin	GO:0061630
WDR48	Ubiquitin	GO:0043130
WDR77	Ubiquitin	GO:1990756
WDR81	Ubiquitin	GO:0070530
WDSUB1	Ubiquitin	GO:0004842
WFS1	Ubiquitin	GO:0031625
WRAP53	Ubiquitin	GO:0031625
WSB1	Ubiquitin	GO:0061630
WWP1	Ubiquitin	GO:0004842;GO:0061630
WWP2	Ubiquitin	GO:0004842;GO:0061630
XBP1	Ubiquitin	GO:0031625;GO:0044389
XIAP	Ubiquitin	GO:0004842;GO:0061630
XRCC5	Ubiquitin	GO:0031625
YOD1	Ubiquitin	GO:0004843;GO:0031625;GO:0061578;GO:0101005;GO:1990380
YWHAE	Ubiquitin	GO:0031625
YWHAZ	Ubiquitin	GO:0031625
ZBTB1	Ubiquitin	GO:0070530
ZC3H12A	Ubiquitin	GO:0004843
ZFAND2B	Ubiquitin	GO:0031593;GO:0036435;GO:0043130
ZFAND6	Ubiquitin	GO:0031593
ZFP91	Ubiquitin	GO:0004842;GO:0061630
ZMYM2	Ubiquitin	GO:0031624
ZNF598	Ubiquitin	GO:0055106;GO:0061630
ZNF675	Ubiquitin	GO:0031625
ZNF738	Ubiquitin	GO:0004842
ZNRF1	Ubiquitin	GO:0004842;GO:0061630
ZNRF2	Ubiquitin	GO:0061630
ZNRF3	Ubiquitin	GO:0004842;GO:0061630
ZNRF4	Ubiquitin	GO:0061630
ZRANB1	Ubiquitin	GO:0004843;GO:0031593;GO:0070530;GO:0101005
ZRANB3	Ubiquitin	GO:0070530
ZSWIM2	Ubiquitin	GO:0004842;GO:0061630
ZSWIM8	Ubiquitin	GO:1990756
ZUP1	Ubiquitin	GO:0004843

## Functions of Ub and Ubl in ER‐ribosomal‐associated QC: Limiting further un/misfolded protein accumulation in the ER

**Fig. 3:**
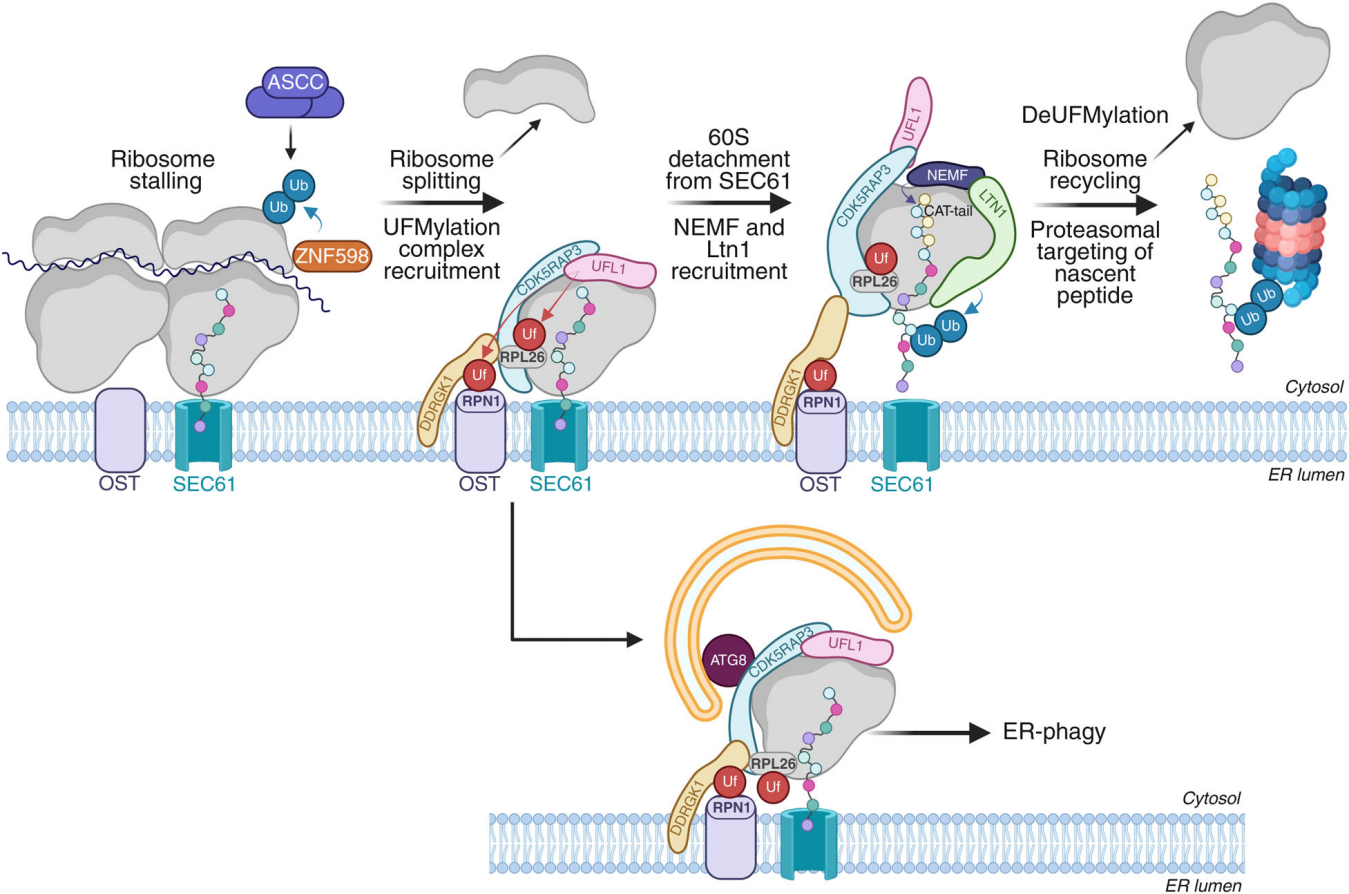
Interplay between ubiquitin, UFM1 and ATG8 in ER‐ribosome quality control. Upon ribosome stalling, K63‐linked ubiquitination of ribosomal proteins by ZNF598 allows recruitment of the ASCC complex, which promotes ribosome subunit splitting. The UFM1 E3 ligase complex (DDRGK1, CDK5RAP3, and UFL1), in coordination with the E2 enzyme UFC1 (not represented here), is then recruited and UFMylates several ribosomal proteins, most prominently RPL26, as well as the OST subunit RPN1. This promotes detachment of the ribosome from the SEC61 translocon and facilitates access of the E3 Ub ligase LTN1 to the nascent peptide. The tRNA‐binding protein NEMF adds a CAT tail to the nascent peptide prior to its K48‐linked ubiquitination by LTN1. DeUFMylation likely promotes dissociation of the UFMylation complex from the ribosome, extraction of the nascent peptide by VCP (not represented here), and its subsequent proteasomal degradation, together with ribosome recycling. In cases of extensive ribosome stalling, UFMylation can alternatively promote ER‐phagy, through a CDK5RAP3‐ and ATG8‐dependent autophagosome recruitment. ASCC, Activating signal cointegrator 1 complex; ATG8, Autophagy‐related 8; CAT, C‐terminal alanine‐threonine; CDK5RAP3, Cyclin dependent kinase 5 regulatory subunit associated protein 3; DDRGK1, DDRGK domain‐containing 1; ER, Endoplasmic reticulum; LTN1, Listerin E3 ubiquitin protein ligase 1; NEMF, Nuclear export mediator factor; OST, Oligosaccharyltransferase; RPL26, Ribosomal protein L26; RPN1, Ribophorin I; Ub, Ubiquitin; UFC1, Ubiquitin‐fold modifier conjugating enzyme 1; UFL1, UFM1‐specific ligase 1; UFM1 (Uf), Ubiquitin fold modifier 1; VCP, Valosin‐containing protein; ZNF598, Zinc‐finger protein 598.

Upon detection of ER stress, several mechanisms are engaged to limit the entry of potentially aberrant proteins into the ER lumen. These include repression of global protein synthesis (via the PERK branch, as discussed earlier), reduction of selected RNA loads through RIDD, as well as ER pre‐emptive QC and ER‐ribosomal‐associated QC (ER‐RQC). Here, we focus on ER‐RQC and the multiple roles of Ub and Ubl in this process (Fig. 3).

Ribosome‐associated QC (RQC) is primarily triggered by ribosome stalling [120, 121, 122], for example when mRNAs lack a stop codon upstream of the polyadenylation site. In such cases, stalling results from the simultaneous presence of poly(A) mRNA within the ribosome decoding center, and the polybasic portion of the nascent polypeptide (a series of lysines) within the exit tunnel, which sterically hinders elongation [123, 124]. In the context of ER‐RQC, ribosome stalling predominantly arises from the blockage of the nascent peptide chain within the translocon. Once stalling occurs, ER‐QC is required to recycle ribosomes, when possible, prevent further synthesis of aberrant polypeptides, and relieve clogging of the SEC61 translocon. ER‐RQC is thus activated upon collision of an upstream translating ribosome with a downstream terminally stalled ribosome. The E3 ubiquitin ligase ZNF598 catalyzes K63‐linked ubiquitination of several proteins of the stalled small ribosome subunit (uS10, eS10, sometimes uS3), thereby enabling recruitment of downstream factors, including the activating signal cointegrator 1 complex (ASCC). The stalled 80S ribosome is then split through ASCC‐dependent mechanisms [125]. Dissociation of the 40S ribosomal subunit allows recruitment of the tRNA‐binding protein nuclear export mediator factor (NEMF) and of the E3 ubiquitin ligase listerin (Ltn1) to the 60S subunit [126]. NEMF further extends the nascent polypeptide chain with a C‐terminal alanine‐threonine (CAT) tail [127], which facilitates extrusion of the peptide from the ribosome exit tunnel and promotes its recognition by LTN1 [128]. LTN1 subsequently catalyzes K48‐linked ubiquitination of the nascent polypeptide on the detached 60S subunit, thereby targeting it for proteasomal degradation [129, 130, 131]. In addition, an LTN1‐independent route has been described, in which recognition of four consecutive alanine residues in the CAT tail enables the E3 ubiquitin ligases KLHDC10 and PIRH2 to ubiquitinate the nascent polypeptide, marking it for proteasomal degradation.

In addition to ubiquitination, several recent studies have uncovered a major role for UFMylation in ER‐RQC (for recent reviews, see [98, 132]). Following ribosome splitting, the UFMylation E3 ligase complex (composed of the ER‐anchored DDRGK1, CDK5RAP3, and UFL1; and acting in cooperation with the E2 UFC1) is recruited to the 60S ribosomal subunit. This complex catalyzes the covalent attachment of UFM1 to K132 and K134 of uL24 (also named RPL26) [133, 134, 135], a large ribosomal subunit located proximal to the translocon, thereby facilitating detachment of the 60S subunit from SEC61. Recruitment of UFL1 occurs independently of NEMF [125], and interaction between UFL1 and NEMF in fact limits or pauses CATylation [136]. UFMylated RPL26 is subsequently recognized by the UFM1‐interacting motif (UFIM) of DDRGK1, which acts as a reader and promotes “clamping” of the UFM1 E3 ligase complex onto the 60S subunit. This clamping blocks both tRNA binding and the peptide exit tunnel, resulting in the displacement of SEC61 and allowing ubiquitination of the nascent aberrant polypeptide by LTN1 [137, 138]. Subsequent deUFMylation likely permits dissociation of the UFL1 complex and access of ANKZF1 [125] and VCP, which releases the ubiquitinated nascent peptide from the tRNA, extracts it from the 60S, and promotes its proteosomal degradation and allows recycling of the 60S ribosomal subunit [136].

Recent data further suggest that UFMylation also participates in canonical eRF1‐dependent translation termination at the ER [137, 138]. Notably, an alternative UFMylation‐dependent pathway for clearance of stalled ER‐associated ribosomes has been reported, although the determinants governing this pathway choice remain unclear. In this route, UFMylation of RPL26 is recognized by the translocon‐associated UFM1 sensor SAYSD1, which, together with the translocon‐associated TRAPP complex, promotes lysosomal clearance of the clogged translocon [127, 139]. UFMylation can also trigger ER‐phagy [101, 140, 141], particularly under conditions of extensive ribosome stalling, likely reflecting saturation of the canonical RQC mentioned above [132]. Beyond RPL26, the UFMylation E3 ligase complex also targets the oligosaccharyltransferase (OST) subunit ribophorin‐1 (RPN1) [101]. CDK5RAP3 possesses dual binding capacity for UFM1 and ATG8 through its shuffled ATG8‐interacting motifs (sAIMs) [140, 142, 143]. Upon overwhelming ribosome stalling, increased UFMylation of substrates such as RPN1 and RPL26 enables CDK5RAP3 interaction with ATG8, promoting autophagosome recruitment and ER‐phagy of selected UFMylated ER subdomains [134, 140, 143]. An additional substrate, NADH‐cytochrome B5 reductase 3 (CYB5R3), is also UFMylated, recognized by UFIM of DDRGK1, and degraded via ER‐phagy, suggesting that UFMylation may function as a broader surveillance mechanism for ER integrity by targeting damaged ER regions and ER‐resident proteins removal [141]. Overall, these studies highlight how Ub, UFM1, and ATG8 function as key regulators of ER‐RQC, collectively orchestrating cellular adaptation to ER proteotoxicity.

## Roles of Ub and Ubl in ER‐associated degradation (ERAD): Expelling the un/misfolded proteins from the ER

As part of the ER stress response, several protein degradation pathways are activated to eliminate misfolded, unfolded or slow‐folding proteins that accumulate in the ER. These complementary processes include ERAD, ER‐lysosomal degradation (ERLAD), as well as ER‐phagy. ERAD is an essential pathway, as illustrated by the lethality observed in mice lacking key components such as HRD1 or SEL1L. Although ERAD is initially known for its role in the removal of misfolded proteins (which will be the focus of this section), ERAD also plays a crucial role in regulating ER lipid composition through control of sterol synthesis enzyme levels, maintaining calcium homeostasis (via regulation of IP3R levels), and modulating the formation of MCS between the ER and other organelles [144]. All of these functions can ultimately influence ER proteostasis.

**Fig. 4:**
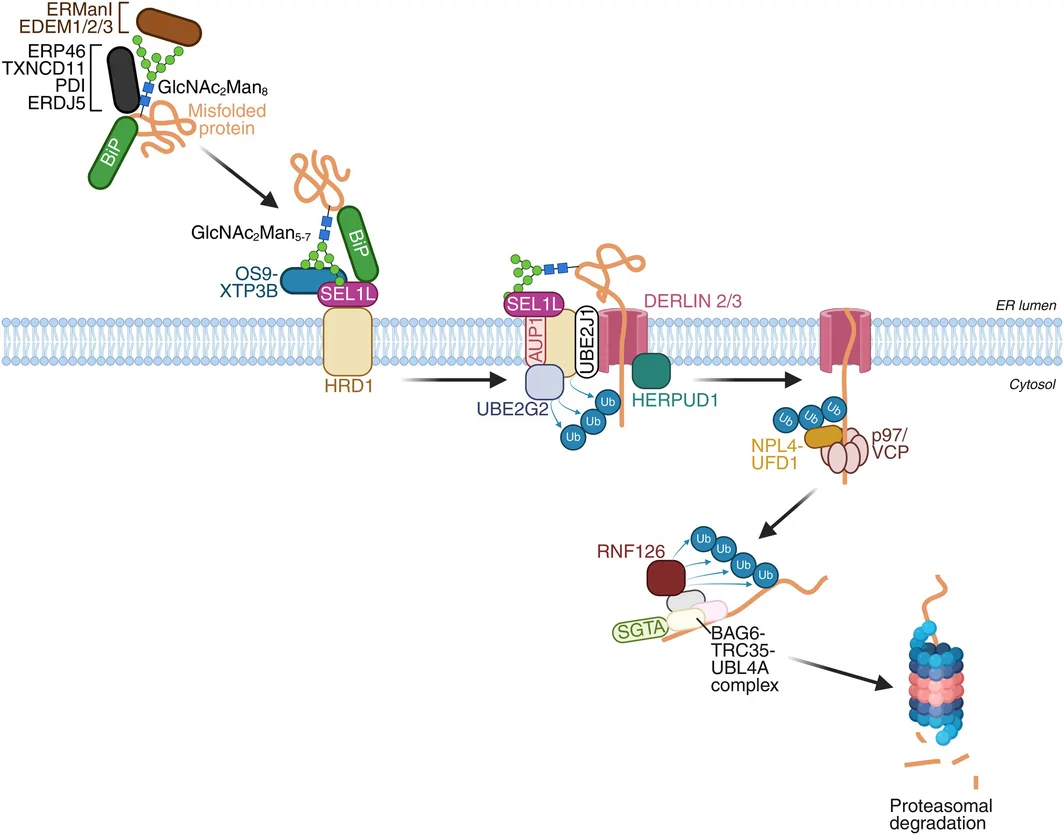
Key steps of ERAD and involvement of ubiquitin. Example for a luminal glycoprotein substrate. Repeated unsuccessful folding attempts result in prolonged exposure of N‐linked glycans to the mannosidases and glucosidases ERManIA, ManIA and EDEM1/2/3 along with PDIs and related proteins (PDI, TXNCD11, ERP46 and ERDJ5), promoting progressive glycan trimming, and reduction of disulfide bonds. This prevents recognition of misfolded substrates by the lectin chaperones calnexin and calreticulin, thereby blocking further folding cycles. In addition, the trimmed glycans (GlcNAc2Man5–7) are recognized by the lectins OS9 and XTP3B, which, together with BiP and SEL1L, deliver the substrate to the E3 Ub ligase HRD1. HRD1 associates with DERLIN 2/3 and additional cofactors such as HERPUD1, AUP1, and E2 Ub‐conjugating enzymes (e.g., UBE2G2 and UBE2J1). The substrate is then retrotranslocated and, as it emerges in the cytosol, is first mono‐ubiquitinated and subsequently modified by K48‐ and K11/K48‐linked polyubiquitin chains, promoting its recognition by the NPL4‐UFD1 complex and VCP‐mediated extraction. SGTA and the BAG6‐TRC35‐UBL4A complex recognize the substrate, preventing aggregation and promoting K48‐linked polyubiquitination by the E3 Ub ligase RNF126, followed by proteasomal degradation. AUP1, Ancient Ubiquitous Protein 1; BAG6, BAG Co‐chaperone 6; BiP, Binding‐immunoglobulin protein; DERLIN 2/3, Der1‐Like Domain Family, Member 1/2; EDEM1/2/3, ER degradation‐enhancing alpha‐mannosidase‐like protein 1/2/3; ER, Endoplasmic reticulum; ERAD, ER‐associated degradation; ERDJ5, Endoplasmic Reticulum DNA J Domain‐Containing Protein 5; HRD1, Hydroxymethylglutaryl‐CoA reductase degradation 1 homolog; HERPUD1, Homocysteine inducible ER protein with ubiquitin like domain 1; ERManI, ER Mannosidase IA; ERP46, ER protein 46; NPL4, Nuclear protein localization 4; OS9, Osteosarcoma amplified 9; PDI, Protein disulfide isomerases; RNF126, RING finger protein 126; SEL1L, Sel‐1 suppressor of Lin‐12‐like 1; SGTA, Small glutamine rich tetratricopeptide repeat co‐chaperone alpha; TRC35, Transmembrane domain recognition complex subunit of 35 kDa; TXNCD11, Thioredoxin domain‐containing 11; Ub, Ubiquitin; UBE2G2/J1, Ubiquitin conjugating enzyme G2/J1; UBL4A, Ubiquitin like 4A; UFD1, Ubiquitin recognition factor In ER‐associated degradation 1; VCP, Valosin‐containing protein; XTP3B, XTP3‐transactivated protein B.

ERAD is inherently a Ub‐dependent process (Fig. 4). In mammals, distinct ERAD pathways operate depending on the nature of the substrate, engaging different Ub E3 ligases and associated cofactors [145, 146]. Despite this diversity, the core steps are conserved. These include the recognition of misfolded proteins within the ER, retrotranslocation across the ER membrane for luminal substrates, or dislocation of transmembrane domains for membrane‐integrated substrates; and ubiquitination by ER‐membrane‐associated E3 ligase complexes as the substrates become exposed to the cytosol. Subsequently, substrates are unfolded and extracted from the ER membrane through the AAA+ ATPase VCP. Further remodeling of the polyubiquitin chains promotes their delivery to the 26S proteasome for degradation. For detailed mechanistic insights into these processes, the reader could refer to recent reviews [144, 145, 147]. Briefly, substrate recognition depends on the presence of a degron (*i.e*., degradation signal) located within one or more regions of the targeted protein. These degrons are detected by one or more recognition cofactors (e.g., BiP, OS9, XTP3B, SEL1L) in the ER lumen, within the ER lipid bilayer, or on the cytosolic side. A single substrate may be recognized by different cofactors and therefore directed to distinct ER‐associated E3 ligase complexes. However, the precise molecular determinants governing substrate discrimination among the various ER E3 ligases remain incompletely understood. ERAD of luminal substrates has been mainly characterized for misfolded soluble glycoproteins [148]. In this context, repeated unsuccessful folding attempts result in prolonged exposure of N‐linked glycans to lectin‐like mannosidase and glucosidases, including ERManIA, ManIA and EDEM1/2/3. EDEM1/2/3 form complexes with protein disulfide isomerase and related factors such as PDI, TXNCD11, ERP46, or ERDJ5 [148, 149, 150, 151, 152, 153]. Some of these partners promote the EDEM1/2/3‐dependent mannose trimming, leading to reduction of disulfide bonds and progressive processing of N‐linked glycans to GlcNAc2Man5–7 structures. As a consequence, misfolded substrates can no longer be recognized by the lectin chaperones calnexin and calreticulin, which bind the higher‐order GlcNAc2Man9 glycoform; thereby preventing further folding cycles. Importantly, mannose trimming generates a glycodegron (an oligosaccharide‐based degron) that is recognized by the lectins OS9 and XTP3B. These lectins are also associated with BiP to detect hydrophobic regions of misfolded proteins and, together with SEL1L, deliver the substrate to the ERAD E3 Ub ligase HRD1. Given the central role of mannose trimming and lectin‐mediated recognition in targeting substrates to the ERAD machinery, this process must be tightly regulated and has been proposed to involve ER stress‐dependent compartmentalisation of its components (reviewed in [148]). Targeting of terminally misfolded luminal substrates that do not display glycodegrons also depends on SEL1L and HRD1, and involves cofactors such as BiP, ERDJ4/5, GRP170, HERPUD1 and EDEM1 for substrate delivery. For membrane protein substrates, the ERAD pathway engaged depends on the localisation of the degron, within the ER lumen, the transmembrane region or the cytoplasmic domain. When a luminal glycodegron is present, degradation typically proceeds via HRD1 complex and relies on ER lectins, as described above. For transmembrane domain‐embedded or cytoplasmic degrons, HRD1 is less commonly engaged and is instead replaced by other ER‐resident E3 ligases, including RNF145/170/185, MARCH6, TRC8, CGRRF1, TMEM129 or GP78. These ligases generally act in conjunction with degron‐recognition cofactors such as ERLIN1/2 or INSIG1/2 (for comprehensive overviews, see [144, 145]). Additional cofactors, including HERPUD1, UBXD8 or UBAC2, contribute to this early ERAD step by promoting assembly and stabilization of ER‐E3 ligase complexes. Nevertheless, many aspects governing degron specificity toward a given ER‐E3 ligase complex (or a subset of such ligases) remain to be elucidated. It is also likely that additional ERAD‐associated E3 ligases will be identified in specific cellular or tissue‐dependent contexts. Following substrate recognition and engagement by an ER‐E3 complex, retrotranslocation into the cytosol must occur. For luminal substrates, this process is thought to involve the formation of a proteinaceous channel composed of the transmembrane domains of HRD1 and DERLIN2/3 [154]. For membrane‐embedded substrates, multiple dislocation mechanisms have been described. For instance, DERLIN1 associates and cooperates with various ER‐E3 ligases, including RNF5 and TMEM129 [155, 156]. In some cases, proteolytic cleavage by proteases such as RHBDL4, SPP or SPC facilitates substrate dislocation [145]. As substrates emerge into the cytosol, exposed hydrophobic regions are protected to prevent aggregation by SGTA and the holdase complex composed of BAG6, UBL4A, and TRC35, which function in coordination with the ER‐resident E3 ligases [157, 158, 159]. ER‐E3 ligases (more than 40 have been identified in mammalian cells, see [145, 146]), together with E2 Ub‐conjugating enzymes (including UBE2J1/2, UBE2G2, UBE2D3, UBE2K, or UBE2L6) and adaptor proteins such as AUP1, catalyze ubiquitin transfer to the substrate. Following an initial priming step (typically monoubiquitination), substrates are polyubiquitinated, mostly through K48‐, K11/K48‐ or K48/K63‐linked chains on exposed lysine residues, and in some cases on serine, threonine or cysteine residues. Polyubiquitinated substrates are subsequently extracted from the ER membrane by VCP, which can be recruited directly to ER‐E3 ligases (e.g., HRD1 or GP78), or indirectly via partners such as DERLIN1/2 or UBXD8. The NPL4‐UFD1 complex recognizes K48‐linked polyubiquitin chains and further facilitates VCP‐mediated extraction. During this process, ubiquitin chains are remodeled through Ub unfolding within the VCP pore and partial deubiquitination by YOD1 [160]. Upon release from VCP, substrates can undergo additional K48‐linked polyubiquitination mediated by the E3 ligase RNF126, in cooperation with BAG6, thereby ensuring efficient targeting to the proteasome for degradation [161].

Selected Ubls may influence ERAD, either directly or indirectly. For instance, the UFMylation machinery contributes to the ERAD of several model substrates, albeit in a cell type‐dependent manner [133, 162]. Moreover, UFMylation of HRD1 enhances its ERAD‐associated activity toward IRE1 [102], and UFMylation has also been implicated in the retrotranslocation of HLA‐A2 [163]. As discussed earlier, SUMOylation regulates XBP1 and ATF6, and may thereby indirectly modulate the expression of ERAD components. Notably, the classical ERAD substrate ΔF508‐CFTR can itself be SUMOylated, which influences its ubiquitination and subsequent proteasomal degradation [164, 165]. In addition, several ERAD machinery components have been identified as putative ISGylation substrates [166], and ISGylation has been reported to negatively regulate proteasomal degradation [167]. However, a direct functional role for ISGylation in ERAD remains hypothetical at this stage.

## Conclusion

An appropriate cellular response to ER proteotoxicity is essential for organismal homeostasis. Ub and Ubl modifications are emerging as versatile regulatory signals that coordinate the timing and engagement of the diverse cellular processes activated during ER stress. Notably, Ub and Ubl also regulate various components of the ISR. For example, PKR modification by ISG15 promotes its activation [168] when SUMOylation could either enhance or repress PKR activity depending on the SUMO isoform [169, 170]. Of note, cell survival of multiple cancer cell types relies on the ubiquitination and subsequent degradation of the ISR kinase HRI by UBA6/BIRC6/KCMF1/UBR4. This dependency on BIRC6 is particularly enriched in cancer cells with a high degree of aneuploidy, for which it could constitute an interesting therapeutic opportunity [171]. This further highlights the broader roles these PTMs play in cellular stress adaptation and underlines the importance of studying them. Beyond their role in protein QC, these PTMs also contribute to the regulation of MCS formation between the ER and other organelles, thereby influencing organelle morphology and function as well as overall ER homeostasis. For instance, during ER stress in melanoma cells, XBP1s promotes the expression of the Ub E3 ligase MARCH5, which contributes at least part to the degradation of MFN2. This, in turn, induces mitochondrial fission and mitophagy, thereby enhancing resistance to cell death [172]. Altogether, a deep understanding of the nature, the timing and the subcellular localization of Ub/Ubl‐mediated regulation during ER proteotoxicity and stress responses more broadly, will likely be critical for the development of future therapeutic strategies targeting diseases associated with cellular stress.

## Methods

### Ubiquitin and ubiquitin‐like pathway gene annotation and transcription factor overlap analysis

Gene Ontology (GO) Molecular Function terms associated with ubiquitin and ubiquitin‐like activities, restricted to *Homo sapiens*, were retrieved from the GO database using the AmiGO2 tool (https://amigo.geneontology.org/). The associated GO terms were manually curated and grouped into functional machineries based on their annotated enzymatic roles (e.g., E1, E2, E3 enzymes, deubiquitinases, autophagy‐related ATG8 machinery). For ubiquitin‐like pathways, individual GO terms were associated with one or more functional machineries, and genes annotated to multiple GO terms were assigned to all corresponding machineries.

Gene‐GO mappings were obtained using the org.Hs.eg.db annotation package, and gene identifiers were harmonized to HGNC symbols. Genes shared between ubiquitin and ubiquitin‐like annotations were assigned exclusively to the ubiquitin‐like category to avoid double counting. In addition, a curated list of autophagy‐related ubiquitin‐like genes was manually added to the ubiquitin‐like gene set. The recently described E1 and E2 enzymes involved in human URMylation were also manually added to the URM1 machinery gene set [173].

Transcription factor target genes were retrieved from ChIP‐Atlas (https://chip‐atlas.org/), and for each transcription factor, the top 1000 ranked target genes were retained for downstream analyses. For each ubiquitin or ubiquitin‐like machinery, transcription factor coverage was calculated as the proportion of machinery‐associated genes present within the corresponding transcription factor target list. Percentages were computed independently for each machinery and each transcription factor. Results are represented as stacked bar plots, illustrating the relative contribution of each transcription factor to the coverage of ubiquitin and ubiquitin‐like functional machineries.

## Conflict of interest

The authors declare no conflict of interest.

## Author contributions

Conceptualization and supervision: EL; Visualization and writing‐original draft: EL; Visualization and writing‐review and editing: EL, MLG, TA; Funding acquisition: EL, MLG, TA.

## Data Availability

All data used to generate Fig. 2 are publicly available, from https://amigo.geneontology.org/ and https://chip‐atlas.org/.
